# TMPRSS2-mediated SARS-CoV-2 uptake boosts innate immune activation, enhances cytopathology, and drives convergent virus evolution

**DOI:** 10.1073/pnas.2407437121

**Published:** 2024-05-30

**Authors:** Bingqian Qu, Csaba Miskey, André Gömer, Robin D. V. Kleinert, Sara Calvo Ibanez, Regina Eberle, Aileen Ebenig, Dylan Postmus, Maximilian K. Nocke, Maike Herrmann, Tabitha K. Itotia, Simon T. Herrmann, Natalie Heinen, Sebastian Höck, Florian D. Hastert, Christine von Rhein, Christoph Schürmann, Xue Li, Ger van Zandbergen, Marek Widera, Sandra Ciesek, Barbara S. Schnierle, Alexander W. Tarr, Eike Steinmann, Christine Goffinet, Stephanie Pfaender, Jacomina Krijnse Locker, Michael D. Mühlebach, Daniel Todt, Richard J. P. Brown

**Affiliations:** ^a^Division of Veterinary Medicine, Paul-Ehrlich-Institut, 63225 Langen, Germany; ^b^European Virus Bioinformatics Center, 07743 Jena, Germany; ^c^Division of Medical Biotechnology, Paul-Ehrlich-Institut, 63225 Langen, Germany; ^d^Department of Molecular and Medical Virology, Ruhr University Bochum, 44801 Bochum, Germany; ^e^Electron Microscopy of Pathogens, Paul-Ehrlich-Institut, 63225 Langen, Germany; ^f^Department of Tropical Disease Biology, Liverpool School of Tropical Medicine, Liverpool L3 5QA, United Kingdom; ^g^Institute of Virology, Charité-Universitätsmedizin Berlin, 10117 Berlin, Germany; ^h^Department of Physical Sciences, Chuka University, 60400 Chuka, Kenya; ^i^Division of Virology, Paul-Ehrlich-Institut, 63225 Langen, Germany; ^j^Department of Cardiology, Medical University Hospital, 69120 Heidelberg, Germany; ^k^Division of Immunology, Paul-Ehrlich-Institut, 63225 Langen, Germany; ^l^Institute for Immunology, University Medical Center of the Johannes Gutenberg University of Mainz, 55131 Mainz, Germany; ^m^Research Center for Immunotherapy, University Medical Center, Johannes Gutenberg-University Mainz, 55131 Mainz, Germany; ^n^Institute for Medical Virology, University Hospital Frankfurt, Goethe University Frankfurt, 60596 Frankfurt am Main, Germany; ^o^Fraunhofer Institute for Translational Medicine and Pharmacology, 60596 Frankfurt am Main, Germany; ^p^German Center for Infection Research, 38124 Braunschweig, Germany; ^q^School of Life Sciences, Faculty of Medicine and Health Sciences, University of Nottingham, Nottingham NG7 2UH, United Kingdom; ^r^School of Life Sciences and National Institute for Health and Care Research, Nottingham Biomedical Research Centre, University of Nottingham, Nottingham NG7 2UH, United Kingdom; ^s^Research Unit Emerging Viruses, Leibniz Institute of Virology, 20251 Hamburg, Germany; ^t^University of Lübeck, 23562 Lübeck, Germany; ^u^Justus Liebig University Geissen, 35390 Giessen, Germany; ^v^German Center for Infection Research, 63225 Giessen-Marburg-Langen, Germany

**Keywords:** SARS-CoV-2 entry, innate immunity, cytolytic responses, viral evolution, host-species range

## Abstract

The accessory protease transmembrane protease serine 2 (TMPRSS2) enhances severe acute respiratory syndrome coronavirus 2 (SARS-CoV-2) uptake into ACE2-expressing cells, although how increased entry impacts downstream viral and host processes remains unclear. To investigate this in more detail, we performed infection assays in engineered cells promoting ACE2-mediated entry with and without TMPRSS2 coexpression. Electron microscopy and inhibitor experiments indicated TMPRSS2-mediated cell entry was associated with increased virion internalization into endosomes, and partially dependent upon clathrin-mediated endocytosis. TMPRSS2 increased panvariant uptake efficiency and enhanced early rates of virus replication, transcription, and secretion, with variant-specific profiles observed. On the host side, transcriptional profiling confirmed the magnitude of infection-induced antiviral and proinflammatory responses were linked to uptake efficiency, with TMPRSS2-assisted entry boosting early antiviral responses. In addition, TMPRSS2-enhanced infections increased rates of cytopathology, apoptosis, and necrosis and modulated virus secretion kinetics in a variant-specific manner. On the virus side, convergent signatures of cell-uptake-dependent innate immune induction were recorded in viral genomes, manifesting as switches in dominant coupled Nsp3 residues whose frequencies were correlated to the magnitude of the cellular response to infection. Experimentally, we demonstrated that selected Nsp3 mutations conferred enhanced interferon antagonism. More broadly, we show that TMPRSS2 orthologues from evolutionarily diverse mammals facilitate panvariant enhancement of cell uptake. In summary, our study uncovers previously unreported associations, linking cell entry efficiency to innate immune activation kinetics, cell death rates, virus secretion dynamics, and convergent selection of viral mutations. These data expand our understanding of TMPRSS2’s role in the SARS-CoV-2 life cycle and confirm its broader significance in zoonotic reservoirs and animal models.

## Significance

Virus tropism is governed by multiple factors at the host-virus interface, determining infection outcomes in individual cells, tissues, and species. We report that transmembrane protease serine 2 (TMPRSS2) enhances severe acute respiratory syndrome coronavirus 2 (SARS-CoV-2) internalization into endosomes, boosting innate immune activation which potentially promotes clearance and is beneficial to the host. In parallel, TMPRSS2-mediated uptake increases virus-induced cytopathology and selects for variants with enhanced immune evasion properties, which may contribute to more severe disease. Together these data highlight an intricate balance between viral uptake efficiency, cell-intrinsic responses, and infection-induced pathology. Furthermore, we report SARS-CoV-2 variant-specific differences in TMPRSS2 dependency, which impact immune activation, cytotoxicity, and virion secretion and may contribute to differences in clinical severity observed between strains.

The severe acute respiratory syndrome coronavirus (SARS-CoV-2) has spread globally since its initial spillover to humans in late 2019 (1). Infection results in a broad variety of clinical outcomes, ranging from asymptomatic to severe pneumonia and associated immune dysregulation that can lead to fatal multisystemic failure (2). The emergence of transmission-enhancing mutations, mainly in the spike glycoprotein (S), has given rise to multiple variants, possibly driven by immune escape from human antibody targeting induced by natural infection and vaccination (3). Omicron (B.1.1.529) is now the dominant circulating variant worldwide, with subsequent replacement of the original BA.1/2 variants with a succession of more transmissible sublineages. B.1.1.529 lineage viruses and their descedants exhibit significant immune escape from neutralizing antibodies induced by first-generation vaccines or prior infection with non-Omicron variants (4), and exhibit milder pathogenesis owing to an altered cell tropism (5).

**Fig. 1.**
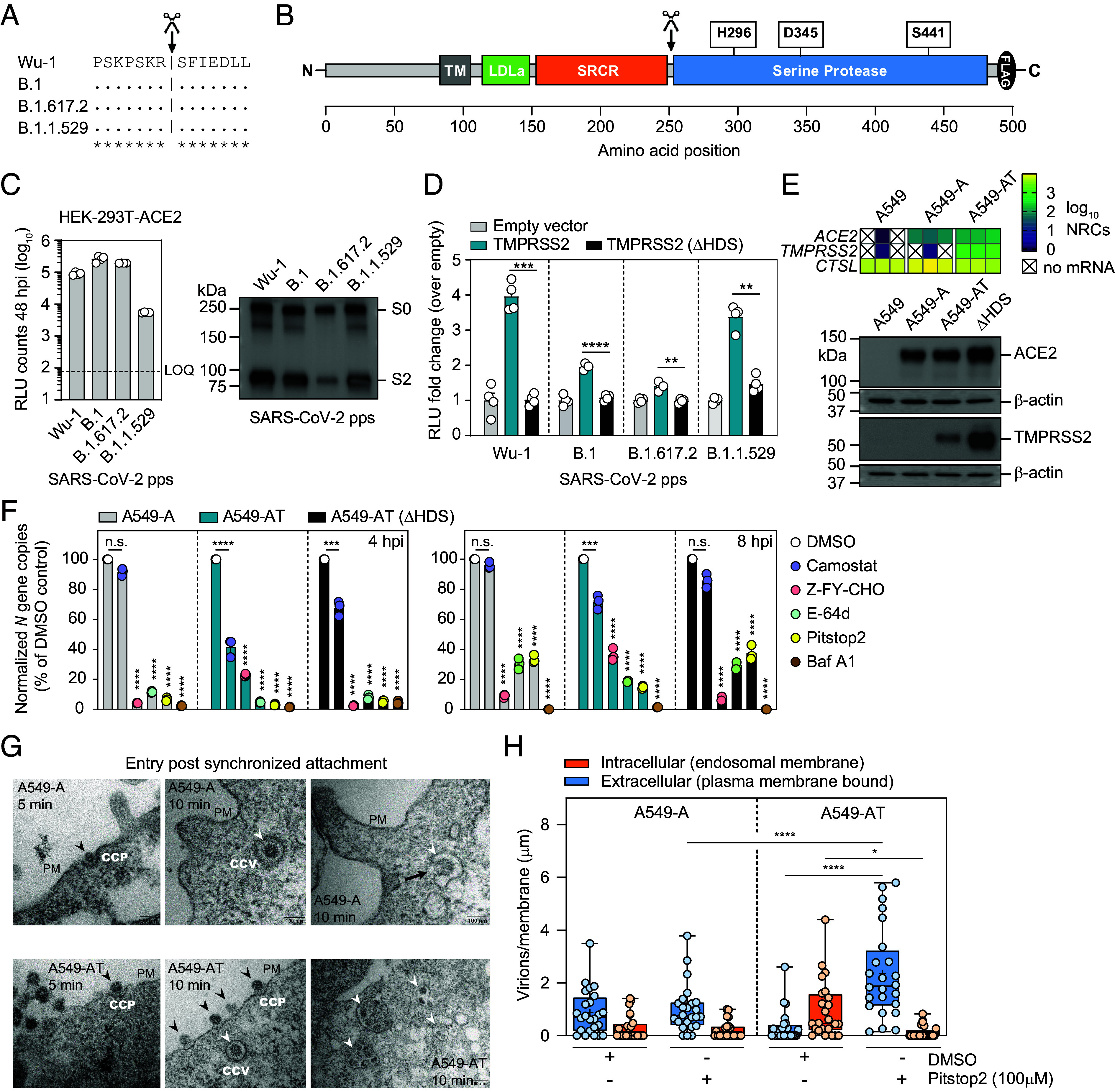
TMPRSS2 enhances SARS-CoV-2 internalization into endosomes. (*A*) Conservation of S2’ site between variants. (*B*) Cartoon of human TMPRSS2 protein domains. Location of catalytic triad residues and autocleavage site (scissors) are highlighted above. (*C*) SARS-CoV-2 pp cell entry of the indicated S variants. *Left* panel: RLU counts at in HEK-293T-ACE2 cells at 48 hpi. RLU: Relative light units. LOQ: limit of quantification. (N = 4 mean ± SEM). *Right* panel: Western blot quantification of S protein expression in SARS-CoV-2 containing supernatants. (*D*) Uptake of the indicated SARS-CoV-2 pps in the presence of TMPRSS2 or TMPRSS2 (ΔHDS). (N = 4 mean ± SEM. **P < 0.01, ***P < 0.001, ****P < 0.0001). (*E*) Human *ACE2* and *TMPRSS2* mRNA (*Top*, N = 3) and protein expression (*Bottom*) in the indicated cell lines. (*F*) Authentic B.1 vRNA levels in the indicated cell lines at 4 hpi (*Left*) and 8 hpi (*Right*) in the presence of the indicated inhibitors, normalized to vRNA levels in DMSO control treated cells. MOI = 0.01. N = 3 (mean ± SEM). (*G*) EM visualization of authentic B.1 virus entry into A549-A and A549-AT cells. Virus uptake by endocytosis is observed at 5 and 10 min in both cell lines, with B.1 binding to CCPs (*Left* and *Middle* panels; black arrowheads). In both cell lines at 10 min, B.1 is observed in CCVs (*Middle* and *Right* panels; white arrowheads) and endocytic compartments (*Right* panels; white arrowheads). In A549-A cells at 10 min (*Top Right*), the black arrow highlights a virus–host membrane fusion event. In A549-AT cells at 10 min (*Bottom Right*) clusters of virions accumulate in endosomal compartments (white arrowheads). PM: plasma membrane; CCV: clathrin-coated vesicle; CCP: clathrin-coated pit. (*H*) Quantification of extracellular and internalized virions. Normalized virion counts per µm/membrane for extracellular plasma membrane–associated virions (blue) or internalized endosomal virions (orange). Infections were performed as in (*G*), in the presence of either DMSO or Pitstop 2 (100 µM), and image quantification was performed at 10 min post temperature shift. MOI = 500. (N = 24 mean ± SEM. *P < 0.05, ****P < 0.0001).

Despite S protein diversity between variants, the entry step represents a conserved point of the SARS-CoV-2 lifecycle. To enter cells, viral S protein engagement of angiotensin-converting enzyme 2 (ACE2) (1, 6) is critical and induces conformational changes in S. Prior to genomic RNA delivery into the cytosol, S cleavage by host proteases at S2’ occurs (Fig. 1*A*). This exposes the viral fusion peptide (FP) and precedes virus–host membrane fusion. Two host proteases with distinct cellular localizations are involved in S-FP exposure. The transmembrane protease serine 2 (TMPRSS2) localizes to the plasma membrane, whereas the low-pH-dependent cysteine protease cathepsin L is expressed in intracellular vesicles. If TMPRSS2 is absent, the entire virus-ACE2 complex is internalized via clathrin-mediated endocytosis (CME) (7) and virus–host membrane fusion occurs in endosomes, facilitated by cathepsin L (8, 9). In contrast, in the presence of plasma membrane–localized TMPRSS2, virus entry is more efficient (6, 10) and virus–host membrane fusion is proposed to occur directly at the cell surface, bypassing CME (11–13).

In humans, TMPRSS2 is expressed in nasal, lung, and gut cells (14). TMPRSS2 belongs to the family of type II transmembrane serine proteases and contains an N-terminal cytoplasmic tail, a transmembrane (TM) domain, a low-density lipoprotein receptor class A domain (LDLa), a scavenger receptor cysteine-rich domain (SRCR), and a C-terminal serine protease domain containing a catalytic triad (H296, D345, S441) which mediates substrate cleavage (Fig. 1*B*). The TMPRSS2 protein can be autoprocessed or processed by other proteases at the R255-I256 junction. While the biological function of TMPRSS2 in the host is not fully understood, protease domain-mediated entry enhancement of a broad range of respiratory viruses is reported, including influenza, HCoV-229E, SARS-CoV, and MERS-CoV (15–18). Therefore, TMPRSS2 represents a promising host target for antiviral interventions and therapeutic targeting by small molecule inhibitors has been reported in vitro and in vivo (6, 19–22).

While viral entry enhancement by TMPRSS2 is well documented, conflicting data exist concerning levels of enhancement for different SARS-CoV-2 variants. Furthermore, visualization of virus uptake via distinct pathways is currently lacking, and the impact of entry efficiency on downstream host and viral processes is unknown. Finally, the contribution of TMPRSS2 entry enhancement to the broad species range of SARS-CoV-2 is undefined. To investigate these knowledge gaps, we engineered HEK-293T cells and A549 lung adenocarcinoma cells to promote ACE2-mediated viral entry in the presence or absence of TMPRSS2, to allow for direct comparison, and infected them with S-bearing pseudoparticles (pps) and authentic viruses respectively. We visualized early authentic viral entry steps with and without TMPRSS2 using electron microscopy (EM). We explored whether ancestral and more recent SARS-CoV-2 variants differ in their TMPRSS2 dependence for entry, and whether entry efficiency impacts downstream virus replication and transcription, virus production, and infected-cell lifespan. Using RNA-sequencing (RNA-seq) of virus-infected cells, we investigated whether TMPRSS2-primed entry boosts host innate immune responses and whether these host responses imprint viral genome evolution. Finally, we asked whether SARS-CoV-2 entry enhancement is a conserved property of TMPRSS2 orthologues from evolutionarily diverse nonhuman species.

## Results

### TMPRSS2 Expression Enhances Virus Internalization.

HEK-293T-ACE2 cells were infected with lentiviral pps decorated with S proteins from ancestral viruses (Wu-1 and B.1 D614G, hereafter B.1) and more recent variants (B.1.617.2 and B.1.1.529) (Fig. 1 *C*, *Left*). Comparison of RLU counts at 48 hours postinfection (hpi) revealed increased cell entry of B.1 and B.1.617.2 pps when compared to Wuhan (Wu-1), with reduced B.1.1.529 entry despite similar levels of S incorporation into pps (Fig. 1 *C*, *Right*). To compare variant-specific entry enhancement rates mediated by TMPRSS2, lentiviral plasmids containing either no transgene (empty), human TMPRSS2, or a catalytically inactive version (ΔHDS) were transduced into HEK-293T-ACE2 cells 48 h prior to infection with pps (Fig. 1*D*). Entry of all S variants was significantly enhanced by TMPRSS2 expression when compared to the catalytically inactive version, albeit to differing degrees. Wuhan pp entry was most enhanced, with sequential reductions observed for B.1 and B.1.617.2 entry. In contrast to some reports, we found B.1.1.529 entry was robustly enhanced by TMPRSS2 expression.

Next, we engineered A549 cell lines to promote SARS-CoV-2 uptake via ACE2 alone (A549-A) or in combination with TMPRSS2 (A549-AT). RNA-seq confirmed minimal or no *ACE2* or *TMPRSS2* expression and high endogenous cathepsin L (*CTSL*) expression in parental A549 cells, while stable expression of transgene mRNAs and proteins were confirmed in engineered cells (Fig. 1*E*). Authentic B.1 infections were then performed in the presence of a panel of entry inhibitors at noncytotoxic concentration (*SI Appendix*, Fig. S1*A*), and internalized viral RNAs (vRNAs) were quantified at 4 and 8 hpi. As expected, across all treatment conditions, vRNAs were consistently higher in A549-AT cells, when compared to A549-A and A549-AT(ΔHDS) cells (*SI Appendix*, Fig. S1*B*). Therefore, to directly compare inhibitor efficacy across the different cell lines, vRNA copies were normalized to the respective DMSO control cells (Fig. 1*F*). Camostat mesylate treatment (TMPRSS2-specific) inhibited entry into A549-AT cells, while Z-FY-CHO treatment (CTSL-specific) was more effective in A549-A and A549-AT(ΔHDS) cells. Of note, endosome acidification inhibitor bafilomycin A1 (Baf A1) and CME inhibitor Pitstop2 effectively inhibited entry into all engineered cells, with Pitstop2 consistently showing more potent inhibition in A549-AT cells (Fig. 1*F*). These data highlight effective pharmacological inhibition of entry associated processes in engineered A549 cells, with distinct inhibition profiles observed when TMPRSS2 is expressed or absent.

We next visualized uptake of authentic B.1 virus into A549-A and A549-AT cells by electron microscopy (EM). To maximize capturing entry events, which are localized and transient, concentrated B.1 (MOI 500) was bound to cells at 4 °C for 1 h, followed by a switch to 37 °C, and images captured at 5 and 10 min. Irrespective of TMPRSS2 expression, in both A549-A and A549-AT cells, virions were observed bound to clathrin-coated pits (CCP) at the cell surface, intracellularly in clathrin-coated vesicles in (CCV), and accumulated in intracellular compartments of likely endocytic origin. (Fig. 1*G*). Quantification at 10 min (DMSO controls) revealed greater numbers of viruses internalized into endosomal compartments in A549-AT cells, when compared to A549-A cells, indicating that TMPRSS2 expression is associated with enhanced virion internalization into intracellular compartments (Fig. 1*H* and *SI Appendix*, Fig. S2). Internalization of virions in both cell lines was reduced by the CME inhibitor Pitstop2 (100 µM), confirming endocytosis of virions occurs with or without TMPRSS2 present. Consistently, in Pitstop2 treated A549-AT cells, virions accumulated in high numbers at the plasma membrane bound to clathrin-coated pits. These data indicate virions are “locked-out” of A549-AT cells upon Pitstop2 treatment, accumulating on the plasma membrane, and highlight the importance of CME in TMPRSS2-mediated entry. Together these data indicate TMPRSS2-mediated entry in engineered A549 cells is associated with increased rates of CME-dependent virion internalization into endosomal/membranous compartments.

### TMPRSS2 Expression Enhances Early SARS-CoV-2 RNA Replication and Secretion in a Variant-Specific Manner.

**Fig. 2.**
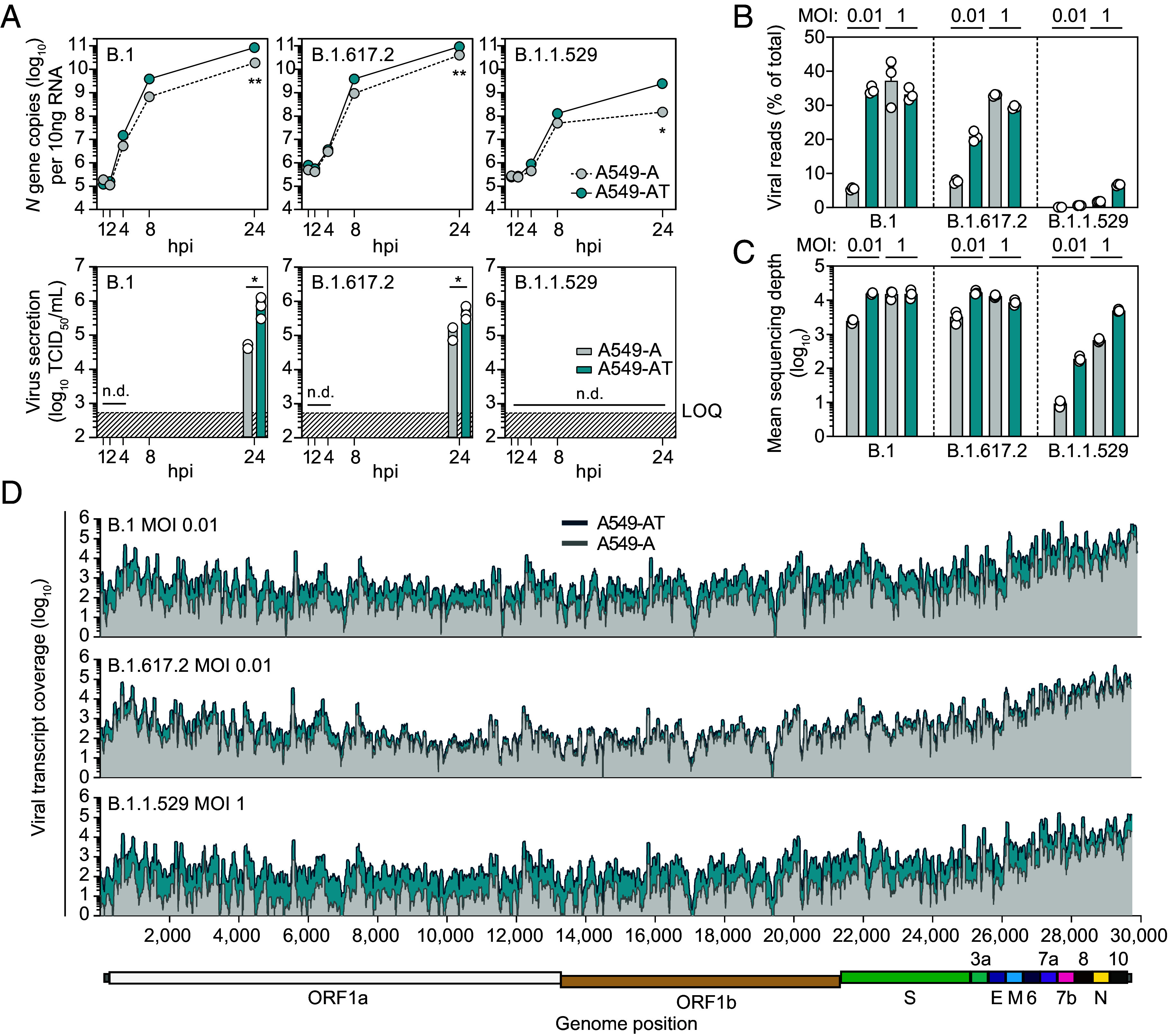
TMPRSS2 expression increases panvariant replication and modulates virus secretion. (*A*) TMPRSS2 expression increases virus replication and secretion rates in a variant-dependent manner. Infections were performed at MOI 0.01 with the indicated SARS-CoV-2 strains, and supernatants and cellular RNAs harvested at 1, 2, 4, 8, and 24 hpi. *Top* panels: Intracellular *N* gene copies determined by RT-qPCR. (N = 3 mean ± SEM). *Bottom* panels: secreted virus in supernatants. TCID_50_. Tissue culture infectious dose 50. (N = 3 mean ± SEM. *P < 0.05, **P < 0.01). LOQ: limit of quantification. n.d.: not detectable. (*B*) Virus mapped reads as a percentage of total reads. Infections were performed with the indicated SARS-CoV-2 strains at low (0.01) and high (1) MOI, with cellular RNAs harvested at 24 hpi for RNA-seq. (*C*) Mean SARS-CoV-2 vRNA sequencing depth. (*D*) Direct comparison of mean viral read coverage across the respective SARS-CoV-2 genomes in A549-A and A549-AT cells. Cartoon directly below represents the relative locations of genome-encoded ORFs.

We next compared early vRNA accumulation and virion secretion profiles in A549-A and A549-AT cells infected with a panel of authentic virus variants (B.1, B.1.617.2, and B.1.1.529). Viral nucleocapsid (*N*) gene copies were quantified by RT-qPCR and virion secretion into supernatants determined by a 50% tissue culture infectious dose (TCID_50_) limiting dilution assay (Fig. 2*A*). Irrespective of the infecting variant, vRNA accumulation was consistently enhanced in A549-AT cells, with differences between cell lines becoming more pronounced at later time points (Fig. 2 *A*, *Upper*). Of note, the steep replication increase observed between 2 and 8 hpi for B.1 and B.1.617.2 was less pronounced (~1.5 to 2 log) for B.1.1.529.

For all variants, no virions were detected above the assay LOQ at 1 to 8 hpi. At 24 hpi, differential virus secretion rates were observed between A549-A and A549-AT cells for B.1 (~17-fold) and B.1.617.2 (~4.5-fold) (Fig. 2 *A*, *Lower*). Irrespective of TMPRSS2 expression, no B.1.1.529 secretion from engineered A549 cells was detected, while robust secretion from VeroE6 cells at 24 hpi was confirmed (*SI Appendix*, Fig. S3). These data demonstrate increased virus entry translates to increased virus replication and secretion for B.1 and B.1.617.2 and confirm reduced TMPRSS2 dependence of B.1.617.2 compared to B.1. They also highlight impaired B.1.1.529 replication in engineered A549 cells, although early replication could still be enhanced by TMPRSS2-mediated uptake. In contrast, early B.1.1.529 secretion was completely abolished in both A549-A and A549-AT cells at 24 hpi.

To investigate early vRNA accumulation rates in more detail, A549-A and A549-AT cells were infected at both low (0.01) and high (1) MOI and vRNA accumulation at 24 hpi determined by RNA-seq. Reads were mapped against the respective viral and human reference genomes, and the ratio of viral reads to total cellular reads calculated (Fig. 2*B* and *SI Appendix*, Fig. S4). At low MOI, a 6.4-fold increase in B.1 vRNAs was observed between A549-A and A549-AT cells, compared to a ~2.7-fold increase for B.1.617.2. At high MOI, minimal differences were observed between B.1 and B.1.617.2 vRNA levels in A549-A and A549-AT cells, indicating saturation. In line with RT-qPCR data and despite abundant ACE2 expression, B.1.1.529 vRNAs were drastically reduced when compared to B.1 and B.1.617.2. However, a ~6.2-fold increase between A549-A and A549-AT cells at low MOI and a ~3.9-fold increase at high MOI confirmed enhanced B.1.1.529 entry mediated by TMPRSS2 is reflected in downstream replication and transcription rates.

Mean sequencing depth (Fig. 2*C*) and read coverage across viral genomes (Fig. 2*D*) highlight differences in early viral replication and transcription rates apparent between A549-A and A549-AT cells. Independent of sequencing depth or infecting strain, these analyses also revealed highly conserved mapping profiles tiled across SARS-CoV-2 genomes, confirming conservation of ORF expression and genome replication kinetics between ancestral virus and more recent variants. Taken together, these data confirm that TMPRSS2 expression enhances infection, and concomitantly, early replication and transcription rates of B.1, B.1.617.2, and B.1.1.529 variants, with B.1.617.2 exhibiting the least TMPRSS2 dependency. In addition, these data point to limits on viral replication and barriers to B.1.1.529 dissemination in A549 cells which are independent of ACE2 expression.

### TMPRSS2-Primed Entry Promotes Early Activation of Cellular Antiviral Programs.

**Fig. 3.**
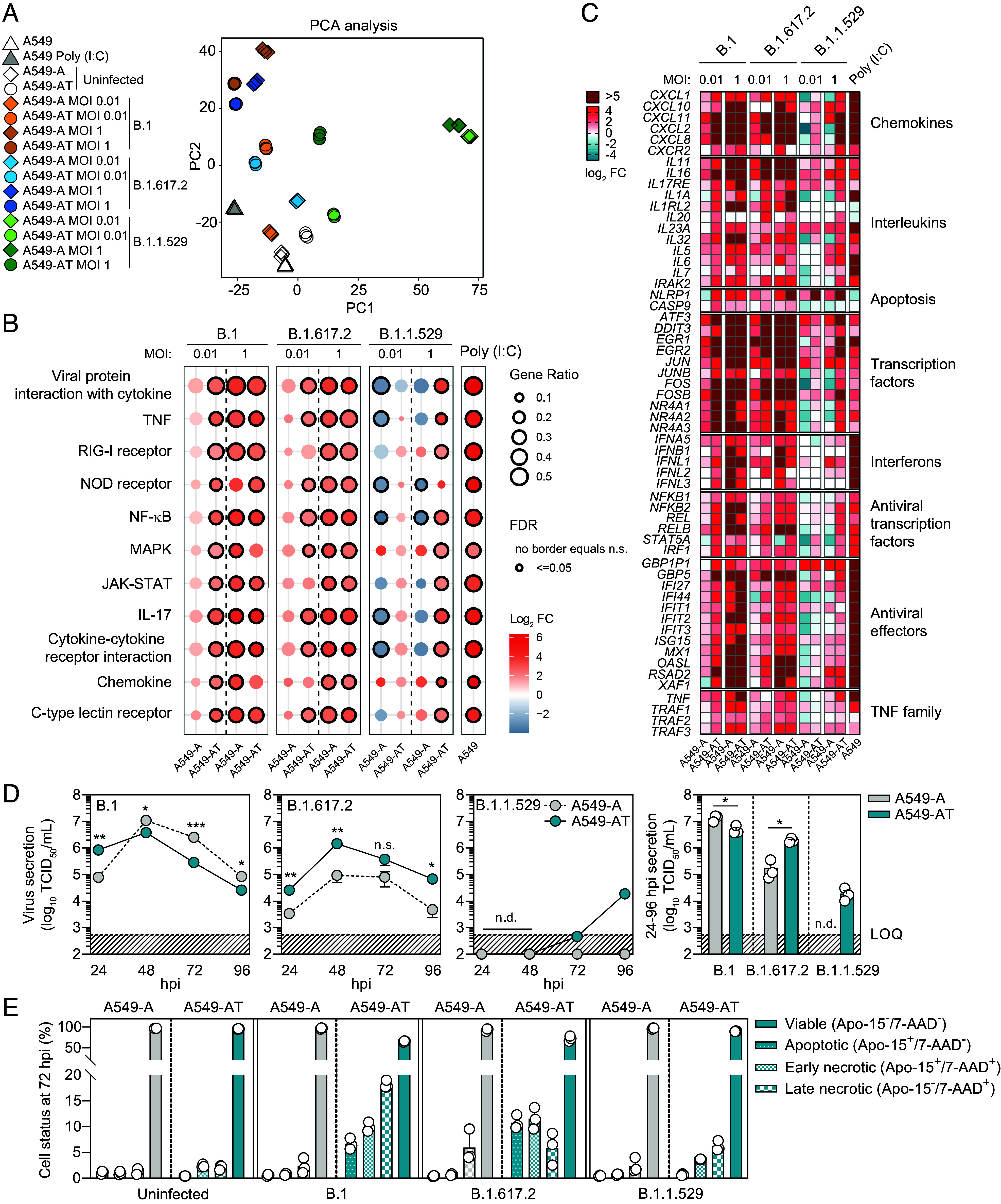
TMPRSS2-mediated entry increases the magnitude of early antiviral responses. (*A*) Principal component analysis (PCA) of the indicated transcriptomes. (*B*) Dot-plot summarizes cell processes and signaling pathways targeted upon infection. FDR: false discovery rate. N = 3. (*C*) Heat map visualizes fold change (log_2_) in mRNA expression for selected host transcripts associated with different cellular processes after SARS-CoV-2 infection of A549-A and A549-AT cells. Fold change is relative to expression levels in the respective uninfected control cell line and represents mean values from N = 3 biological replicates. (*D*) Virus production rates in the indicated cell lines (MOI 0.01). *Left* panel: Virion production kinetics 0 to 96 hpi. *Right* panel: Cumulative virus production. (N = 3 mean ± SEM. *P < 0.05, **P < 0.01, ***P < 0.001. n.s.: not significant). LOQ: limit of quantification. n.d.: not detectable. (*E*) Cell viability of the indicated cell lines at 72 hpi, determined by flow cytometry. (N = 3 mean ± SEM).

More efficient viral entry concomitantly increases initial viral replication and transcription. At 6 hpi, more vRNAs were detected in engineered A549 cells compared to liver epithelial Huh7.5.1 and Huh7.5.1-T cells, due to higher ACE2 expression (*SI Appendix*, Fig. S5*A*). However, fold changes in vRNA levels between 6 and 24 hpi were enhanced in Huh7.5.1 cells when compared to A549 cells, likely due to the absence of endogenous interferon (IFN) induction (*SI Appendix*, Fig. S5*B*). Based on these data, we reasoned that enhanced vRNA levels in A549-AT cells would concomitantly impact the magnitude of early cell-intrinsic antiviral responses. Statistical analyses of host-cell gene dysregulation at 24 hpi identified differentially expressed genes (DEGs) induced upon SARS-CoV-2 infection of A549-A or A549-AT cells (*SI Appendix*, Fig. S6). To confirm the parental cell line’s ability to mount antiviral responses, we also included parental A549 cells transfected with the dsRNA mimic Poly(I:C). Principal component analyses (PCA) of individual transcriptomes revealed tight clustering of biological replicates, with segregation of individual clusters dependent on infection status, presence or absence of TMPRSS2 expression, infecting strain and MOI (Fig. 3*A*) Infection-induced transcriptional dysregulation modulated a range of cell-intrinsic processes and canonical signaling pathways. We performed KEGG pathway analyses and confirmed that significant infection-induced changes in the cellular transcriptome mainly affected processes associated with immunity and inflammation (Fig. 3*B*). Targeted cellular processes were conserved across strains, with significant activation proportional to vRNA abundance.

To visualize differential patterns of DEG induction, we zoomed in on selected subsets of genes which were significantly induced upon infection (Fig. 3*C*), confirming a subset by RT-qPCR (*SI Appendix*, Fig. S7). Induction rates of antiviral transcription factors (TFs), type I and III IFNs, and antiviral effector transcripts were broadly proportional to vRNA levels in cells. At low MOI, minimal induction by B.1 or B.1.617.2 infection was observed in A549-A cells, while robust induction was apparent in A549-AT cells. Similar induction patterns were observed for genes encoding chemokines, interleukins, apoptosis factors, tumor necrosis factor (TNF)-related transcripts and TFs including acute response genes and nuclear receptors (Fig. 3*C*). This effect was more pronounced for B.1 than B.1.617.2. The distinction between cell lines was lost at high MOI, where viral replication and transcription presumably reached saturation and robust induction was seen in both cell types. For B.1.1.529, robust induction was only observed at high MOI in A549-AT cells. Together these data indicate that early suppression of the IFN-system occurs below certain replication threshold, presumably mediated by virally encoded immune antagonists. At low MOI, this threshold is overrun by TMPRSS2-mediated entry, resulting in enhanced delivery of viral genomes to the cytosol to seed initial replication and concomitant early innate immune activation. Furthermore, these data suggest that the reduced B.1.1.529 tropism for A549 cells is not due to an inability to antagonize the IFN system, as broad induction of antiviral responses, which suppresses replication and abrogates early virion release, was not observed. Indeed, at high MOI, B.1.1.529 infection of A549-AT cells, replication overshoots this threshold and innate immunity is activated.

### TMPRSS2 Expression Modulates Virus Production Kinetics and Cell Lifespan.

To investigate the effects of enhanced uptake, replication, and innate immune activation mediated by TMPRSS2 on downstream virus production kinetics, we monitored virus secretion rates in time-course experiments from 24 to 96 hpi. Distinct viral egress profiles were observed for individual variants, which differed between A549-A and A549-AT cells (Fig. 3*D*). B.1 release from A549-AT cells was initially higher at 24 hpi, but this pattern was reversed at later time points, with enhanced production from A549-A cells. In contrast, B.1.617.2 production was consistently higher in A549-AT cells compared to A549-A cells. B.1.1.529 production was either completely abolished or impaired, remaining undetectable in supernatants from A549-A cells and detectable above the assay LOQ only at 96 hpi in A549-AT cells.

SARS-CoV-2 is a cytolytic virus, killing infected cells. For all variants, higher rates of syncytia coupled with increased numbers of apoptotic/necrotic cells were observed in A549-AT cells when compared to in A549-A cells (*SI Appendix*, Fig. S8 and Fig. 3*E*). Interestingly, B.1 production in A549-AT cells was reduced at 48 to 96 hpi when compared to A549-A. Consistent with this observation, B.1 infected A549-AT cells exhibited the most pronounced cytopathic effect (*SI Appendix*, Fig. S8) and enhanced levels of virus-induced necrosis at 72 hpi, followed by B.1.617.2, and B.1.1.529 (Fig. 3*E*). These data point to a reduced lifespan for highly permissive A549-AT cells infected with B.1 which results in reduced cumulative virion production (Fig. 3 *D*, *Right*). Together these data highlight differences in the temporal dynamics of particle production and virus-induced cell death which are modulated by the presence or absence of TMPRSS2 expression, and which also differs between variants.

Leveraging in vivo omics data from our previous study (23), we observed depletion of lung *ACE2* and *TMPRSS2* transcripts in B.1 infected hamster lung at 96 hpi, compared to uninfected control animals (supplementary *SI Appendix*, Fig. S9*A*). This pattern likely reflects infection-induced death of permissive (*Ace2*^+^) and highly permissive (*Ace2*^+^*Tmprss2*^+^) cells, mirroring what we observe in vitro in Fig. 3*E*. Indeed, contrasting upregulation of *Ctsv* (homologous to *CTSL*) chemokines, interleukins, antiviral TFs, and effector transcripts was observed. Visualizing accessory protease expression in hamster lung single-cell (sc)RNA-seq data revealed endogenous *Tmprss2* expression was largely confined to alveolar type 1 and 2 (AT12) cells, while *Ctsv* expression was generally higher and more broadly expressed (*SI Appendix*, Fig. S9 *B* and *C*). Together these analyses validate aspects of our in vitro model in vivo and confirm *Tmprss2* entry enhancement is likely confined to a subset of AT12 cells in hamster lung.

### Innate Immune Activation Status Modulates the Frequencies of Specific Viral Mutations in Nsp3.

**Fig. 4.**
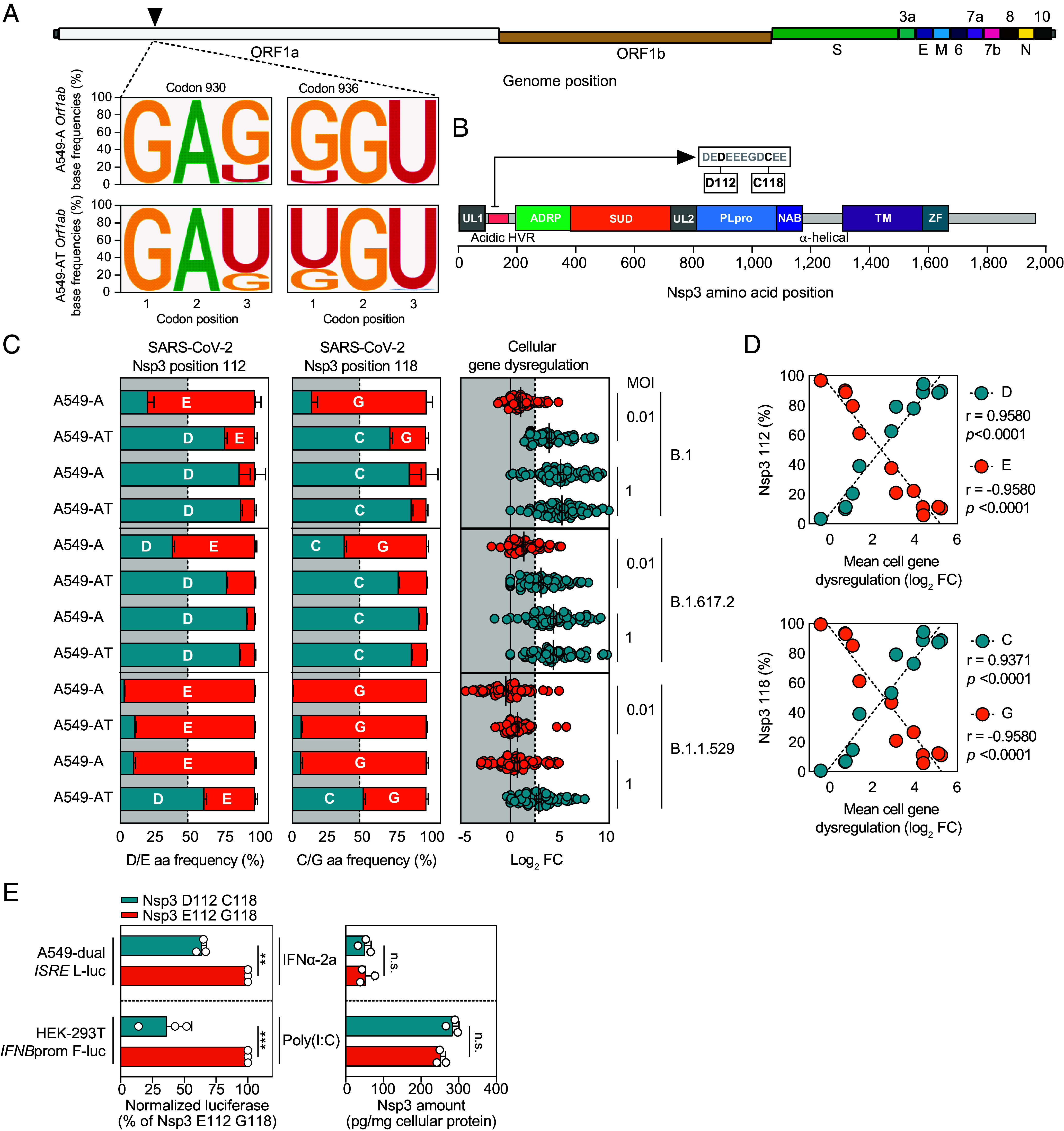
Convergent mutational switching at two Nsp3 residues confers enhanced interferon antagonism. (*A*) Codon switching in Orf1a/Orf1ab. Sequence logos depict codon-triplet base frequencies at codons 930 and 936 for B.1 infections at low MOI. *Top* plots: A549-A infections. *Bottom* plots: A549-AT infections. (*B*) Cartoon depicting the protein domains of Nsp3. UL: ubiquitin-like domain, HVR: hypervariable region, ADRP: ADP-ribose phosphatase, SUD: SARS-unique domain, PLpro: papain-like protease, NAB: nucleic acid binding domain, TM: multipass transmembrane domain, and ZF: zinc finger motif. Zoom of an 11 amino acid stretch of the Nsp3 acid HVR highlighting positions D112 and C118 is positioned above. (*C*) Amino acid frequency plots for the indicated viruses and cell lines highlight shifts in dominant Nsp3 residues 112 (*Left*) and 118 (*Middle*), compared to cellular gene dysregulation under the same conditions (*Right*). (*D*) Residue frequencies at Nsp3 112 (*Top*) and 118 (*Bottom*) are significantly correlated to cell gene dysregulation. (*E*) Phenotypic characterization of Nsp3 mutants. *Left* panel. Normalized luciferase induction in the indicated cell lines after Nsp3-EGFP fusion protein encoding plasmid transfection and subsequent treatment with PolyI:C or IFNα2a (N = 3 mean ± SEM. **P < 0.01, ***P < 0.001). *Right* panel. Quantification of EGFP expression in HEK-293 T and A549-dual cells (N = 3 mean ± SEM. n.s.: not significant).

Upon transmission, fixation of mutations in RNA virus populations can occur through founder effect or selective sweeps. As the dynamics of early innate immune activation differed between A549-A and A549-AT cells, we hypothesized that the contrasting selective environments in which early viral replication occurs could influence the frequencies of mutations in viral populations. Consequently, we analyzed nonsynonymous single nucleotide variants (nsSNVs) in viral reads from our panel of infected cell lines. Relative to the viral consensus sequence, nsSNVs with frequencies >3% were limited to a handful of sites (*SI Appendix*, Fig. S10). Interestingly, codons 930 and 936 located in Orf1a/Orf1ab exhibited exchanges in dominant codon types (GAG<>GAU and GGU<>UGU, see Fig. 4*A* for B.1 MOI 0.01 example) which were correlated with TMPRSS2 expression, reproducible between three biological replicates and detected in all three variants in parallel.

Orf1a/Orf1ab are synthesized as polyprotein(s) which are posttranslationally cleaved by host and viral proteases into multiple mature proteins. At codons 930 and 936, nsSNVs correspond to amino acid exchanges located in nonstructural protein 3 (Nsp3), a large multifunctional protein containing multiple distinct domains (24) (Fig. 4*B*). In engineered A549 cells, E112 and G118 amino acids were dominant in the viral population when host antiviral responses were minimally activated, while D112 and C118 mutations became dominant when an innate immune activation threshold was breached (Fig. 4 *C*, *Far Right* plot compares host cell gene dysregulation of the panel presented in Fig. 3*C*). Indeed, residue frequencies at these two positions were significantly correlated to the magnitude of cellular gene induction (Fig. 4*D*). Strikingly, this property was conserved in ancestral virus and VOCs, pointing to evolutionary conservation of the viral acidic HVR due to a shared function between strains (Fig. 4*B*).

### Selected Nsp3 Mutations Confer Enhanced Interferon Antagonism.

We hypothesized that the observed switches in Nsp3 acidic HVR residues may provide a selective advantage in cells where innate immunity is activated. Consequently, we transfected luciferase reporter cell lines with plasmids encoding Nsp3-EGFP fusion proteins representing both variants (E112/G118 and D112/C118), prior to treatment with either IFN or transfection with PolyI:C (Fig. 4 *E*, *Left*). Under both conditions, cells transfected with Nsp3-EGFP D112/C118 exhibited consistently reduced induction of luciferase production compared to Nsp3-EGFP E112/G118 transfected cells, while intracellular EGFP expression levels remained consistent (Fig. 4 *E*, *Right*). These data indicate that the selected D112/C118 mutations observed by NGS confer a selective advantage to the virus in cells where innate immunity is induced by enhancing antagonism of cell-intrinsic antiviral responses.

### Evolutionary Conservation of the TMPRSS2 Transmembrane Domain and Serine Protease Catalytic Triad.

**Fig. 5.**
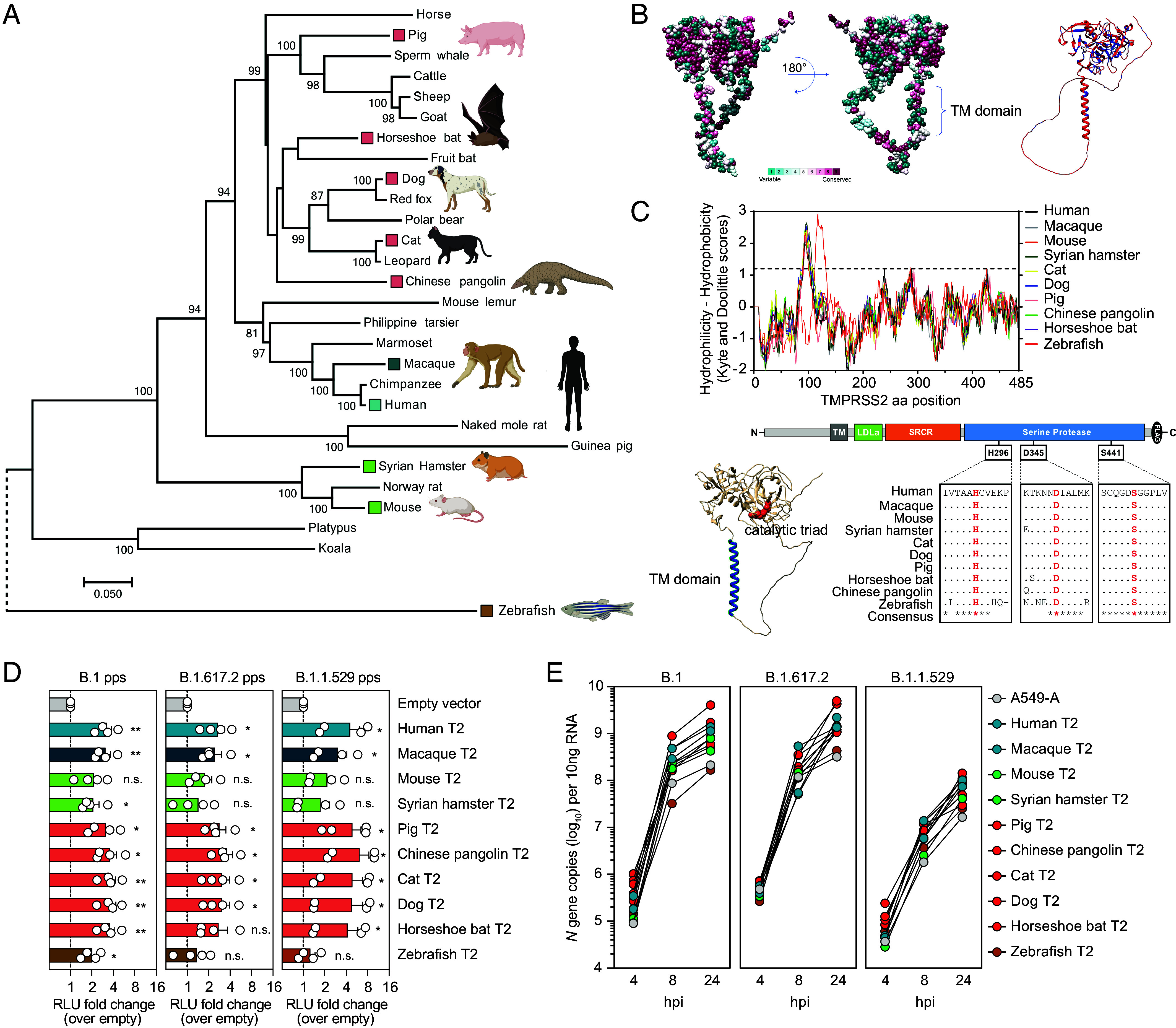
TMPRSS2 proteins from diverse mammals enhance SARS-CoV-2 uptake. (*A*) Phylogenetic tree depicts the evolutionary relationships of *TMPRSS2* coding sequences. Branch lengths are proportional to nucleotide substitutions per site, as determined by the scale bar. Significantly supported groupings (>70%) are labeled at internal nodes and species selected for further investigation are highlighted. (*B*) *Left* panels. Amino acid variability in mammalian TMPRSS2 orthologues is superimposed onto the predicted human TMPRSS2 structure. *Right* panel. Amino acid differences (red) and conservation (blue) between mammalian and zebrafish TMPRSS2 orthologues are superimposed onto the predicted zebrafish TMPRSS2 structure. (*C*) Conservation of transmembrane domains and catalytic triad residues in humans and nonhuman species. *Top* panel. Hydrophobicity plotting identifies a single transmembrane domain in humans and nonhuman species. *Bottom Left* panel. Transmembrane domain (blue) and catalytic triad residues (red) highlighted on the predicted human TMPRSS2 structure. TM. Transmembrane. *Bottom Right* panel. Amino acid alignment confirms conservation of serine protease catalytic triad residues in humans and nonhuman species. (*D*) SARS-CoV-2 pp entry into HEK-293T-ACE2 cells expressing the indicated TMPRSS2 orthologues, normalized to infection rates in empty vector transduced cells. T2: TMPRSS2. (N = 4 mean ± SEM. *P < 0.05, **P < 0.01, n.s.: not significant) (*E*) Viral replicative kinetics in A549-A cells transduced with TMPRSS2 orthologues from 10 species. A549-A cells were transduced with equal amounts of p24-enveloped pps encoding each TMPRSS2 orthologue, prior to infections with B.1, B.1.617.2, and B.1.1.529 viruses. For all viruses, *N* gene copies were determined at time points 4, 8, and 24 hpi.

On the host side, evolutionary conservation of critical ACE2 binding site residues determines the species range of SARS-CoV-2 (25, 26). However, whether TMPRSS2 orthologues from diverse species exhibit evolutionary conservation of entry enhancement remains unknown. To explore this broader question, we first performed phylogenetic analyses of selected *TMPRSS2* encoding sequences from 25 placental mammals, highlighting humans, established animal models, proposed zoonotic reservoirs, and human companion animals. In addition, marsupial and zebrafish sequences were also included for rooting (Fig. 5*A*). These data revealed the evolutionary relationships of *TMPRSS2* gene orthologues are broadly consistent with reported groupings of eutherian mammals (27), although rodents were antecedent to the main placental grouping. Focusing on the nine mammal species highlighted in Fig. 5*A*, primary amino acid residue conservation was mapped onto the predicted human TMPRSS2 structure (28) (Fig. 5 *B*, *Left*), revealing conserved and variable regions in a structural context. The predicted zebrafish TMPRSS2 structure was used as a scaffold to visualize amino acid residues conserved between mammals and zebrafish (Fig. 5 *B*, *Right*). Despite high levels of amino acid divergence, both within mammals and between mammals and zebrafish, absolute conservation of a hydrophobic single-pass transmembrane domain and serine protease catalytic triad residues were observed (Fig. 5*C*). These data confirm that TMPRSS2 proteins from genetically diverse species exhibit conservation of key protein domains necessary for viral spike S2’ cleavage, as both membrane localization and protease activity are important for entry enhancement.

### TMPRSS2 Orthologues from Diverse Species Enhance SARS-CoV-2 Entry.

To investigate whether SARS-CoV-2 entry enhancement is mediated by evolutionarily divergent TMPRSS2 orthologues, lentiviral plasmids encoding each TMPRSS2 protein from the nine mammalian species and zebrafish were transduced into HEK-293T-ACE2 cells 48 h prior to infection with SARS-CoV-2 pps (Fig. 5*D*). Panvariant entry enhancement was mediated by TMPRSS2 proteins of all tested species when compared to empty vector control transduced cells. Consistent with their phylogenetic relatedness (Fig. 5*A*), comparable entry-boosting phenotypes were observed for TMPRSS2 orthologues from primates (human and macaque) and Laurasiatherian mammals (carnivores, ungulates, bats). Enhancement mediated by antecedent rodent lineage TMPRSS2 orthologues (mouse and hamster) were reduced; however, mRNA expression of both of these orthologs was also reduced in HEK-293 T cells (*SI Appendix*, Fig. S11). Highly divergent zebrafish TMPRSS2 mediated minimal uptake boosting despite equivalent mRNA expression.

To augment these data, A549-A cells were transduced with nonhuman TMPRSS2 orthologues 48 h prior to infection with authentic virus, and early vRNA accumulation and virion secretion profiles compared to A549-A cells (Fig. 5*E* and *SI Appendix*, Fig. S11). Consistent with Fig. 2*A*, early B.1.1.529 RNA replication was impaired and the impact of TMPRSS2 expression on vRNA accumulation was most pronounced at 24 hpi. Irrespective of the infecting variant, vRNA accumulation was consistently enhanced in A549-A cells coexpressing mammalian TMPRSS2 orthologues at 24 hpi, when compared to A549-A cells and A549-A cells expressing zebrafish TMPRSS2, and largely mirrored the species-specific enhancement rates observed with SARS-CoV-2 pps, although differences were observed. Therefore, in both systems, TMPRSS2-mediated enhancement by evolutionarily diverse mammalian orthologues was observed, highlighting this cofactor’s contribution to SARS-CoV-2 circulation and dissemination in nonhuman reservoirs and animal models.

## Discussion

Cell entry of all tested SARS-CoV-2 variants was enhanced by TMPRSS2. Wuhan pp entry was highly dependent on TMPRSS2, with successive reductions in entry enhancement observed for B.1 D614G and B.1.617.2 pps, highlighting progressive selection for reduced TMPRSS2 dependency in pre-Omicron pandemic waves. In contrast, B.1.1.529 pp entry was robustly enhanced by TMPRSS2 expression, conflicting with previous reports that B.1.1.529 cell entry has reduced TMPRSS2 dependency compared to B.1.617.2 (29–31). In our hands, B.1.1.529 demonstrated reduced pp entry to HEK-293T-ACE2 cells and lower virus replication in engineered A549 cells. However, enhanced entry and intracellular vRNA accumulation were mediated by TMPRSS2 expression, respectively. Despite robust detection of vRNAs in engineered A549 cells, B.1.1.529 secretion was completely abolished in A549-A cells, and severely impaired or delayed in A549-AT cells (Figs. 2*A* and 3*E*). This altered cellular tropism partially masks the TMPRSS2 dependency of B.1.1.529. Together, these data demonstrate a range of TMPRSS2 dependencies for divergent strains and an altered A549 cell tropism for B.1.1.529, which may mirror its reported altered tissue-tropism (32). These data point to either an absence of uncharacterized B.1.1.529-specific dependency factors or the presence of B.1.1.529-specific restriction factors in A549 cells, which act independently from ACE2 receptor expression.

As expected, pharmacological inhibition of B.1 virus entry with either camostat mesylate (TMPRSS2-specific) or Z-FY-CHO (Cathepsin L-specific) showed distinct efficacy profiles in A549-A and A549-AT cells. E64-d inhibits cysteine proteases in a nonspecific manner. As the virus genome encodes multiple distinct cysteine proteases, it is likely that the dual E64-d inhibition seen in both A549-A and A549-AT cells represents targeting of virally encoded cysteine proteases rather than host Cathepsin L. Pitstop2 targets the clathrin terminal domain to inhibit CME, while vATPase proton pump inhibitor Baf A1 inhibits endosome acidification. Enhanced inhibition by Pitstop2 in A549-AT cells gave us the first hint that CME may be important for TMPRSS2-mediated entry.

We confirm that ACE2-mediated SARS-CoV-2 infection is enhanced in cells expressing TMPRSS2, which localizes at the plasma membrane https://www.proteinatlas.org/ENSG00000184012-TMPRSS2. As S cleavage by TMPRSS2 does not require low pH, it is widely reported that TMPRSS2-assisted virus–host membrane fusion occurs at the plasma membrane. In its absence, viruses are internalized, and virus–host membrane fusion occurs in endosomal membranes after S cleavage by cathepsins. Indeed, CTSL is expressed intracellularly in lysosomes, endosomes, and the Golgi Apparatus https://www.proteinatlas.org/ENSG00000135047-CTSL and requires low pH for function. Our EM analysis of B.1 cell entry identifies surface-bound viruses internalizing through CCPs, and internalized viruses in CCVs, in both A549-A and A549-AT cells (Fig. 1*G*). In A549-AT cells we observed enhanced rates of virion internalization and accumulation in endocytic compartments, when directly compared to A549-A cells (Fig. 1*H*). We confirm by quantification of EM images that Pitstop2 is more potent at inhibiting virion internalization into A549-AT cells. In these experiments, Pitstop 2 effectively locks the virus out of A549-AT cells at 10 min, resulting in virion accumulation on the plasma membrane and indicating CME is a relevant cellular process associated with TMPRSS2-mediated entry (Fig. 1*H* and *SI Appendix*, Fig. S2). It is possible that our EM profiling may have missed surface entry events due to their rapid and transient nature. However, we do not observe internalized electron-dense condensates representing nucleocapsid-bound genomes adjacent to the plasma membrane, which would be expected if surface entry had occurred. The limitations of our experimental system do not allow us to make definitive statements about the distinction between surface versus endosomal routes of entry, which is widely reported for coronaviruses. Nonetheless, these snapshots of SARS-CoV-2 entering engineered A549 cells record the initial molecular interactions occurring at the virus–host interface and provide insights into early SARS-CoV-2 uptake. While outside the scope of this study, our EM observations are the subject of ongoing investigations that aim to analyze cell-type-dependent differences in TMPRSS2-mediated uptake at higher resolution, and in more authentic cell-types.

Our data confirm less efficient entry concomitantly decreased early viral replication, transcription, and secretion. However, at these reduced levels, the virus was able to partially counteract cellular antiviral responses, and innate immunity was not robustly activated. These data confirm SARS-CoV-2 can spread under the radar of innate immunity below a certain replication threshold. Extrapolating these in vitro data to real-life infections, we propose a model whereby highly permissive *ACE2*^+^*TMPRSS2*^+^ cells become an early reservoir supporting rapid viral spread to less permissive *ACE2*^+^ cells. While accelerated virus-induced apoptosis and necrosis occur in highly permissive cells *ACE2*^+^*TMPRSS2*^+^ cells, delayed cellular intrinsic immunity inadvertently leads to an extended period of viral secretion from less permissive *ACE2*^+^ cells. Schuler et al. identified that *TMPRSS2* expression was highest in alveolar epithelial type I cells (AT1) and ciliated cells and increased with aging in humans and mice (33). Indeed, hamster lung profiling identifies enriched *TMPRSS2* expression in AT12 cells and depletion of lung *TMPRSS2* transcripts after infection (*SI Appendix*, Fig. S9). This is consistent with a study demonstrating foci of infection are confined to regions where ACE2 and TMPRSS2 proteins colocalize: bronchioles and alveoli (34). Together, our data shed light on how COVID-19 may initiate and spread, with subtly distinct virus production and innate immune activation profiles associated with *ACE2*^+^ and *ACE2*^+^*TMPRSS2*^+^ expressing cells. Therapeutic targeting of both TMPRSS2 and cathepsins is reported (6, 21, 22, 35, 36), although differential expression levels in specific cell types will affect drug responsiveness. Indeed, in hamster lungs, *Tmprss2* expression was almost exclusively confined to pulmonary AT12 cells while *Ctsv* cell-type expression was generally higher and more broad (*SI Appendix*, Fig. S9*C*). Whether drug candidates can target *TMPRSS2-*expressing cells and efficiently prevent viral entry and spread to *TMPRSS2*-negative cells in vivo is unclear, although simultaneous targeting TMPRSS2 and cathepsins may prove synergistic (37), maximizing potency, and reducing dissemination.

Our data recorded reproducible shifts in viral Nsp3 HVR mutation frequencies which were conserved between ancestral virus and more recent variants, and correlated to the magnitude of host antiviral gene dysregulation determined by entry efficiency. The glutamic/aspartic acid (E/D) rich region in Nsp3 is intrinsically disordered and performs a currently unknown function, although E/D rich proteins are reportedly involved in DNA/RNA mimicry, metal-ion binding, and protein:protein interactions (24). Our experimental investigations indicate that the observed E112D and G118C mutations are selected for upon robust activation of innate immunity due to their enhanced ability to antagonize the IFN system and highlight an immunosuppressive function for the Nsp3 acidic hypervariable region. In the absence of robust innate immune responses, residues D112 and C118 confer a fitness cost and are selectively deleterious: E112 and G118 are dominant in the population. In contrast, when robust immune activation occurs, D112 and C118 are selectively advantageous, promoting improved evasion from host targeting, and their population frequencies move toward fixation.

More broadly, we observed panvariant entry enhancement of SARS-CoV-2 by TMPRSS2 protein orthologues from diverse species, in part mediated by evolutionary conservation of serine protease catalytic triad residues and plasma membrane incorporation. Orthologues from the mammalian Laurasiatheria clade (carnivores, ungulates, bats) and the Euarchontoglire subclade incorporating primates (humans and macaque) exhibited consistent pp entry enhancement. Reduced pp enhancement of antecedent rodent orthologues (mouse and hamster) was observed, although this may be due to reduced expression of these proteins in cells of human origin. This trend was also evident with authentic virus vRNA accumulation at 24 hpi, with consistent vRNA enhancement observed in the presence of mammalian TMPRSS2 orthologues. Together, our data confirm TMPRSS2 represents a broad enhancer of initial infection at the virus–host interface, with diverse mammal species exhibiting the potential to shape the continuous evolution of SARS-CoV-2. Highly divergent zebrafish TMPRSS2 was generally unable to significantly enhance virus uptake, indicating regions outside the conserved transmembrane domain and catalytic triad can also contribute uptake enhancement.

In summary, our data demonstrate differences in TMPRSS2 requirements for entry between SARS-CoV-2 variants correlate with differences in downstream activation of innate immunity after viral entry, which are coupled to vRNA levels in infected cells. TMPRSS2 therefore represents an important modulator of downstream cellular responses to infection. Our EM images indicate TMPRSS2-uptake enhancement is associated with increased virion internalization and depends on CME, which requires further investigation. These data also identify a previously unappreciated inverse relationship between increased efficiency of cellular entry mediated by TMPRSS2, and accelerated rates of apoptosis and necrosis which modulate virus production rates in a variant-specific manner. The footprints of differentially activated host responses are recorded in viral genomes and manifest as switches in dominant amino acids at two Nsp3 residues which confer enhanced immune evasion. Finally, we show that TMPRSS2-mediated entry enhancement is a conserved property of gene orthologues from diverse mammals, including zoonotic reservoirs and experimental models.

## Materials and Methods

### Generation of Stable Cell Lines.

ACE2 and TMPRSS2 gene orthologues used in this study were downloaded from Ensembl or GenBank and chemically synthesized (IDT). The human ACE2 gene was ligated into the lentiviral pTsin-IRES-Puromycin vector via restriction digestion cloning. C-terminal FLAG-tagged TMPRSS2 orthologues and the human ΔHDS mutant were ligated into the lentiviral pWPI-BLR vector (Addgene) via restriction digestion or using HiFi Builder (New England Biolabs). Inserts were confirmed by Sanger sequencing (Eurofins). Lentiviral plasmids were packaged into pseudoparticles (pps) via triple-plasmid cotransfection into HEK-293T cells. Plasmids pTsin or pWPI, psPAX2 (encoding HIV-1 gag/pol) (Addgene), and pMD2.G (encoding VSV-G) (Addgene) were cotransfected in equimolar amounts using Lipofectamine 3000 (Invitrogen). Supernatants containing lentiviral pps were harvested at 24- and 48-h post sodium butyrate induction (10 mM, 6 h) and pooled. Cellular debris was removed by passing pps through 0.45 µM filters, and filtered supernatants were stored at −80 °C prior to use. For generation of stable cell lines, parental A549 cells were first transduced with pseudotyped lentiviral vectors encoding ACE2, followed by a two-week selection with puromycin (2 µg/mL; Sigma-Aldrich). The resulting A549-A cells were either stored at −150 °C or further transduced with pseudotyped lentiviral vectors encoding human TMPRSS2 or the TMPRSS2 ΔHDS mutant, followed by blasticidin selection (20 µg/mL; Fisher Scientific) for two-weeks to generate A549-AT and A549-AT(ΔHDS) cells. Similarly, parental Huh7.5.1 cells were transduced with pseudotyped lentiviral vectors encoding human TMPRSS2 and blasticidin selected to generate Huh7.5.1-T cells.

### SARS-CoV-2 Infections.

All infections were performed under biosafety level 3 conditions. Cells were inoculated with virus in serum-free DMEM for 1 h at 37 °C with gentle shaking of plates every 15 min. After infection, cells were washed three times in PBS and complete DMEM added. For inhibitor treatments, cells were preincubated with camostat mesylate (10 µM; SML0057 Sigma-Aldrich), Z-FY-CHO (10 µM; HY-128140 MedChemExpress), E-64d (10 µM; sc-201280 Santa Cruz Biotechnology), or Pitstop2 (10 µM; ab120687 Abcam) for 1 h prior to infection. After inoculation, these compounds were re-administered in fresh medium. Bafilomycin A1 (Baf A1) (1 µM; Santa Cruz Biotechnology) was added for 1 h pretreatment only due to high cytotoxicity (*SI Appendix*, Fig. S1*A*). For evaluating enhancement of infection by TMPRSS2 orthologues from different species, A549-A cells (5 × 10^5^/well) were seeded in 24-well plates and transduced with equivalent titers of lentiviral pps encoding TMPRSS2 orthologues, determined by p24 capsid ELISA. At 48 h posttransduction, cells were infected with SARS-CoV-2 B.1, B.1.617.2, or B.1.1.529 at MOI 0.01.

### SARS-CoV-2 pps.

SARS-CoV-2 pps were produced by cotransfecting HEK-293T cells with plasmids encoding HIV-1 gag/pol, rev, firefly luciferase, and the respective SARS-CoV-2 S gene, using Lipofectamine 2000 (Invitrogen). SARS-CoV-2 pps were harvested, concentrated by ultracentrifugation, and stored at −80 °C. HEK-293T-ACE2 cells (3 × 10^3^/well) were seeded in 384-well plates and transduced with lentiviral vectors encoding human, mutant, and nonhuman TMPRSS2 orthologues at an MOI of 1, normalized to RNA copies in supernatants. At 48 h posttransduction, cells were infected with SARS-CoV-2 pps. At 48 hpi, entry efficiency was determined via addition of Britelite plus luciferase substrate (Perkin Elmer) using a Tecan Spark luminescence reader (Tecan).

### Transmission Electron Microscopy.

A549-A and A549-AT cells (5 × 10^5^/well) cells were seeded onto coverslips in 24-well plates. Plates and concentrated B.1 virus stocks were precooled to 4 °C before use. Cells were incubated with B.1 virus (MOI 500) at 4 °C for 1 h, allowing synchronized attachment. After 1 h, inoculates were removed and replaced with complete medium prewarmed to 37 °C, and plates shifted to a 37 °C incubator. Infections were stopped at 5- and 10-min post temperature shift by addition of 1/3 volume of 16% paraformaldehyde (#15710 Electron Microscopy Sciences) directly to the media and stored at 4 °C overnight, followed by addition of 2.5% EM-grade glutaraldehyde (G5582 Sigma-Aldrich) in PBS for 30 min at room temperature. After fixation, cells were postfixed with 1% OsO4 in PBS, followed by contrasting with 2% uranyl acetate in water. For embedding in epoxy resin (45359-1A-F, Sigma-Aldrich), cells were dehydrated with increasing concentrations of ethanol, followed by infiltration with increasing concentrations of epoxy resin diluted in ethanol, before infiltration with pure resin and polymerization at 65 °C for 48 h. The hardened monolayer was retrieved from coverslips and re-embedded longitudinally in resin blocks, which were trimmed, and thin transversal sections (70 nm) cut with a 45° diamond knife (Diatome). Sections were placed onto electron microscopy grids and were postcontrasted with 2% uranyl acetate in ethanol and 0.2% lead citrate. Imaging was performed using a Jeol 1400 Flash operated at 120 kV and equipped with an Xarosa camera.

### RNA-Seq.

Cellular RNAs were isolated using TRIzol reagent (ThermoFisher Scientific) and quality checking performed using an Agilent Bioanalyzer. Polyadenylated RNAs were isolated with the NEBNext Poly(A) mRNA module (New England Biolabs) and a modified protocol of the NNSR priming method (38) was used to produce cDNA libraries (39). Sequencing was performed on an Illumina NextSeq550 instrument with an 86 base, single-end setting, using a custom sequencing program. Raw Fastq files were quality- and adapter-trimmed with fastp (40). Mapping to the hg38 human assembly, or to the corresponding viral genomes and gene-level read counting were performed with STAR (41) using default settings. We used the DESeq2 R package (42) for differentially expressed gene (DEG) analysis according to the following workflow. (https://master.bioconductor.org/packages/release/workflows/vignettes/rnaseqGene/inst/doc/rnaseqGene.html). KEGG pathway analyses were performed with clusterProfiler (43) and Pathview packages in R. Bulk and scRNA-seq data from B.1 infected hamsters were generated as previously described (44).

## Supplementary Material

Appendix 01 (PDF)

## Data Availability

RNA-seq data generated in this study have been submitted to the NCBI SRA database (accession number PRJNA868604) (45). All other data are included in the manuscript and/or *SI Appendix*.
