# Supplementary material for: TMPRSS2-mediated SARS-CoV-2 uptake boosts innate immune activation, enhances cytopathology, and drives convergent virus evolution
Source: Proc Natl Acad Sci U S A. 2024 May 30;121(23):e2407437121. doi: 10.1073/pnas.2407437121 (PMC11161796; doi:10.1073/pnas.2407437121)
Supplement: Supplementary file 1 — Appendix 01 (PDF) [file pnas.2407437121.sapp.pdf]

PNAS

Supporting Information for

TMPRSS2-mediated SARS-CoV-2 uptake boosts innate immune activation, enhances cytopathology and drives convergent virus evolution

Bingqian Qu, Csaba Miskey, André Gömer, Robin D.V. Kleinert, Sara Calvo Ibanez, Regina Eberle, Aileen Ebenig, Dylan Postmus, Maximilian K. Nocke, Maike Herrmann, Tabitha K. Itotia, Simon T. Herrmann, Natalie Heinen, Sebastian Höck, Florian D. Hastert, Christine von Rhein, Christoph Schürmann, Xue Li, Ger van Zandbergen, Marek Widera, Sandra Ciesek, Barbara S. Schnierle, Alexander W. Tarr, Eike Steinmann, Christine Goffinet, Stephanie Pfaender, Jacomina Krijnse Locker, Michael D. Mühlebach, Daniel Todt, Richard J.P. Brown

Corresponding author: Richard J.P. Brown
Email: Richard.Brown@rub.de

This PDF file includes:

Supporting text
Figures S1 to S11
SI References

Supporting Information Text
Supplementary materials and methods

Cell lines
A549 and Huh7.5.1 cells were maintained in Dulbecco's modified Eagle's medium (DMEM) (Invitrogen) supplemented with penicillin-streptomycin, 1 mM L-glutamine (Invitrogen) and 10% fetal bovine serum (FBS) (Invitrogen). HEK-293T-hACE2 cells were grown in complete DMEM medium supplemented with zeocin (50 µg/mL; InvivoGen). For VeroE6 cells (ATCC CRL-1586), FBS was reduced to 5%. A549-dual cells (InvivoGen) were maintained in DMEM medium supplemented with 10% FBS, 2 mM L-glutamine, penicillin-streptomycin, 10 µg/mL blasticidin and 100 µg/mL zeocin. A HEK-293T reporter cell line carrying an F-luc reporter under the control of the *IFNß* promotor element was generated with a commercially acquired lentivirus (FencisBIO) and maintained in complete DMEM medium supplemented with Hygromycin (0,1mg/mL; Carl Roth).

Viruses
The B.1 isolate (MUC-IMB1, GenBank accession LR824570) was provided by the Institute for Microbiology, Bundeswehr. The Delta B.1.617.2 isolate (20A/452R) was imported from the Robert-Koch-Institut. The Omicron B.1.1.529 BA.1 isolate (FFM-SIM0550/2021, GenBank accession: OL800702) (1) was provided by Goethe University Frankfurt. Virus stocks were propagated on VeroE6 cells and titers determined on VeroE6 cells using a $TCID_{50}$ limiting dilution assay.

Virus concentration
Unconcentrated supernatants harvested from virus inoculated VeroE6 cells were centrifuged at 3,000 ×*g* for 10 minutes at 4°C to remove cell debris. Supernatants were transferred to 5 Vivaspin 20 columns (100 kD cutoff, Sartorius) and centrifuged at 3,000 ×*g* for 10-15 minutes at 4°C until only 5% of the original volume remained in the upper chamber. Concentrated supernatants were resuspended in the Vivaspin column and pooled for use. Viral titers of unconcentrated virus, concentrated virus, and column flow-through were determined by $TCID_{50}$ assay.

$TCID_{50}$ endpoint assay
For virus titrations, 3×10^4 VeroE6 cells per well were seeded in 96-well plates. The next day, a series of 1:10 dilutions of virus stocks or supernatant from infection experiments were prepared by serially diluting 30 µL of sample with 270 µL serum-free DMEM medium in a U-bottom 96-well plates, prior to transferring to seeded VeroE6 cells. Infected cells were incubated for 4 days at 37°C. Cytopathic effects in each well were identified using a phase contrast light microscope and documented. $TCID_{50}$/mL values were computed using a $TCID_{50}$ calculator.

EM quantification
Electron micrographs were captured using a Jeol 1400 Flash transmission electron microscope operating at an acceleration voltage of 120 kV. For each condition, 24 representative micrographs were acquired at the same magnification. Inclusion criteria were that images contained at least one virion. Selected images were then compiled into stacks for subsequent analysis using Fiji software (2). Manual annotations were performed to measure the length of cellular membranes and to identify intracellular virions (defined as electron-dense particles with an approximate diameter of 60-180nm located within cellular compartments such as clathrin-coated pits or other membranous subcellular structures) or attached to the cell surface membrane. Identification criteria included the presence of electron dense nucleocapsids and/or characteristic viral spikes. To allow for direct comparison between conditions, the virion counts were normalized to the length of the cellular surface membrane in the image. Intracellular and membrane-bound events were compared between cell lines and treatments, and expressed as virion counts per membrane micrometer.

P24 ELISA
To determine p24 concentrations in SARS-CoV-2 pp preparations, a commercially available sandwich ELISA kit was used (ELV-HIVP24, RayBiotech). Briefly, SARS-CoV-2 pp containing supernatants were diluted 1:1 with assay diluent and transferred to an anti-p24 antibody coated microtiter plate in quadruplicates. Absorbance values from tetramethylbenzidine-based detection

were averaged and p24 amounts were calculated. For all SARS-CoV-2 pps, equivalent amounts (125 pg of p24 protein) were lysed prior to Western blotting.

PrestoBlue cell viability assay

A549-A and A549-AT cells (6×10^4) were seeded in 96-well plates and treated with Camostat, Z-FY-CHO, E-64d, Pitstop2 or Bafilomycin A1 at 1:3.2 serially diluted concentrations (0.1; 0.32; 1; 3.2; 10; 32 µM) for 24 hours in 37°C incubator. At the end of treatments, PrestoBlue reagent (A13261, Invitrogen) was freshly 1:10 diluted in culture medium. Cells were incubated with diluted PrestoBlue for 30 minutes at 37°C. Absorbance at 570 nM was read using an ELISA reader (Sunrise Tecan) and normalized to the 600 nM values used as a reference wavelength. Values were further normalized to percentages compared to vehicle DMSO treatment (100%).

Poly(I:C) transfection

High molecular weight (HMW) Poly(I:C) (InvivoGen) was mixed with P3000 (2 µl/µg Poly(I:C)) and Lipofectamine 3000 (2 µl/µg Poly(I:C)) (Invitrogen) in Opti-MEM medium for 15 minutes at room temperature. A549 and Huh7.5.1 cells were incubated with the transfection complex to deliver Poly(I:C) at final concentration of 2 µg/mL. Cells we incubated for 24 hours at 37°C post Poly(I:C) transfection, prior to RNA extraction.

Western blot analysis

Cells or SARS-CoV-2 pps were washed with pre-cooled PBS and lysed in radio-immunoprecipitation assay (RIPA) buffer supplemented with protease inhibitor cocktail (Cell Signaling Technology) for 30 mins on ice. Lysates were centrifuged at 960×*g* for 10 mins at 4°C. Supernatants were harvested for western blot and protein contents were determined by BCA assay (ThermoFisher Scientific) or p24 ELISA as described above. Equal amounts for each sample were mixed with 4× protein loading buffer (200 mM Tris-HCl [pH = 6.8], 400 mM DTT, 8% SDS, 0.4% bromophenol blue, 40% glycerol), heated for 5 mins at 98°C, loaded onto a 4-20% pre-cast SDS gel (Bio-Rad) and resolved by SDS-PAGE. Proteins were subsequently blotted onto a PVDF membrane, which was further blocked with 5% blotting-grade milk or bovine serum albumin in TBST buffer for 1 h. The membrane was incubated overnight at 4°C with anti-ACE2 (1:1,000; #38241 Cell Signaling Technology), anti-TMPRSS2 (1:1,000; HPA035787 Sigma-Aldrich), anti-SARS spike glycoprotein antibody (1:1,000; ab273433 Abcam) or anti-β-actin (1:1,000; Abcam) antibodies with gentle rocking. Washed membranes were subsequently incubated with horseradish peroxidase (HRP) coupled anti-mouse and anti-rabbit IgG(H+L) secondary antibodies (1:3,000, Cell Signaling Technology). Bound antibodies were detected with ECL Plus Detection substrate (GE Healthcare) and visualized using a ChemiDoc Imaging System (Bio-Rad).

RT-qPCR

Cellular RNAs were isolated using a Direct-zol RNA Miniprep Plus Kit (Zymo Research) according to the manufacturer's instructions. RNA concentrations were quantified using a NanoDrop 2000 spectrophotometer (ThermoFisher Scientific) and 500 ng RNA was reverse transcribed into complementary DNA (cDNA) using a PrimeScript II 1st Strand cDNA Synthesis kit (TaKaRa) or a QuantiNova Reverse Transcript Kit (Qiagen) according to the manufacturer's instructions. RT-qPCR of cDNA samples were performed using 2× Rotor-Gene SYBR Green PCR Mastermix (Qiagen) and run on a Rotor-Gene Q real-time PCR thermocycler (Qiagen).

For quantification of SARS-CoV-2 *N* and *RdRp* gene copies, a 319 bp fragment containing partial *N* and *RdRp* genes was cloned into pcDNA3.1Zeo (Thermo Scientific) to generate a pcDNA3.1Zeo-*N-RdRp* plasmid for *in vitro* transcription (New England Biolabs). RNA amounts were quantified and 1×10^{12} copies of RNA were reverse transcribed. The cDNA product was 1:10 serially diluted and used as standards for both genes. For relative quantification of human genes, calculations were performed using the $\Delta\Delta^{Ct}$ method and fold changes were calculated using the $2^{-\Delta\Delta Ct}$ method. For human genes, *GAPDH* was used for normalization. Validated primer pairs were taken from Primer3 (https://primer3.ut.ee/) or from (3). For *TMPRSS2* genes from diverse species, TMPRSS2-encoding nucleotide sequences were aligned, and a conserved region identified for amplification. Equivalent amplification efficiencies for all *TMPRSS2* gene orthologues were then confirmed and absolute quantification was performed by RT-qPCR using a serially diluted plasmid standard.

Flow cytometry
A549-A and A549-AT cells (2.5×10^6) were infected with B.1, B.1.617.2 or B.1.1.529 virus at an MOI of 0.01. At 72 hpi, cells were trypsinized, pelleted and re-suspended in PBS. After washes, cells were fixed with 4% paraformaldehyde (Carl Roth) for 30 mins, pelleted, washed, and re-suspended in FACS buffer (1% FBS in PBS). Cell suspensions were stained with Apotracker Green (BioLegend) and 7-AAD (BioLegend) for 30 mins. Stained cells were washed and analyzed by flow cytometry (FACSymphony, BD Biosciences) and resulting data analyzed using FlowJo v10 software.

Nsp3 reporter assay
An expression plasmid encoding an Nsp3-EGFP fusion protein (D112 C118) was acquired from Addgene (4). In-Fusion cloning (Takara) of two overlapping fragments generated by PCR was used to generate an Nsp3 -EGFP plasmid containing E112 and G118 mutations. Plasmids pNsp3-EGFP (E112 G118) or pNsp3-EGFP (D112 C118) were validated by Sanger sequencing.

For the A549-dual reporter cell-line, 1.5×10^5 cells were plated in 24-well plates and the next day either left untransfected or transfected with 0.5 µg pNsp3-EGFP (E112 G118) or pNsp3-EGFP (D112 C118) plasmids using TransIT-LT1 reagent (Mirus). At 48 hours post transfection, cells were rinsed with PBS twice, trypsinized, centrifuged at 300×g for 5 minutes, counted and resuspended at 2.8×10^5 cells/mL in fresh medium. A total of 180 µL of cell suspension (5×10^4 cells) was added per well, followed by 20 µL of medium or pre-diluted IFNµ-2A (working concentration 1,000 U/mL, PBL) was added into 96-well plates. The mixture was incubated at 37°C for 18 hours. 20 µL of medium sample was used for Lucia luciferase assay by adding 50 µL of QUANTI-Luc 4 solution (InvivoGen). End-point relative luminescence units (RLU) were determined with a 2 second delay time and 10 second reading time in a luminometer (Berthold). For the HEK-293T-IFNß-Fluc reporter cell-line,1×10^5 cells were seeded in Poly-L-Lysine coated 24-well plates and either left untransfected or transfected the next day with 0.5µg pNsp3-EGFP (E112 G118) or pNsp3-EGFP (D112 C118) plasmids using Lipofectamine P3000 (Thermo Fisher). At 24 hours post transfection, cells were washed with PBS twice and fresh media was applied. Afterwards, cells were mock transfected or transfected with Poly(I:C) as described above. At 72 hours post Poly(I:C) treatment, firefly luciferase activity was determined with a Plate Luminometer (Berthold) as described previously (5).

EGFP ELISA
7.5×10^5 A549-dual and HEK-293T cells were plated in 6-well plates, and either left untransfected, or transfected with 2.5 µg pNsp3(E112 G118)-EGFP or pNsp3(D112 C118)-EGFP plasmids. 48 hours later, cells were rinsed with PBS twice, lysed in 100 µL of cell extraction buffer PTR in the kit (ab171581, Abcam). The lysates were centrifuged at 18,000×g for 20 minutes at 4°C. 50 µL of supernatant samples were used for EGFP ELISA assay according to the manufacturer's instructions. To determine amounts of total cellular protein, 1 µL of the same samples were also subjected to BCA protein assay (ThermoFisher). Nsp3(E112 G118) and Nsp3(D112 C118) expression levels were normalized to total cellular protein (pg/mg).

Phylogenetic analysis
TMPRSS2 sequence alignment followed by phylogenetic analysis using the maximum likelihood composite approach implemented under the GTR+I+Γ model was performed using MEGA7 software (6). The analysis incorporated 28 sequences and 1,569 nucleotide positions were included in the final dataset. The significance of groupings was determined using the bootstrap approach with 500 replicates.

TMPRSS2 structures
Structural predictions for TMPRSS2 proteins were downloaded from AlphaFold (7). Evolutionary conservation scores for individual amino acid residues in mammalian orthologue protein alignments were projected onto the predicted human TMPRSS2 protein structure using ConSurf (8) with default settings. Mapping mammalian-zebrafish conservation onto zebrafish TMPRSS2 was performed using UCSF Chimera (9).

Hydrophobicity analysis

Amino acid translations for TMPRSS2 orthologues were pasted in the ProtScale tool in Expasy (https://web.expasy.org/protscale). Hydrophobicity (Hphob) scores for each amino acid were calculated using the Kyte and Doolittle algorithm (10).

Viral diversity analysis

Viral reads were initially mapped against the respective viral reference sequences followed by consensus sequence generation using the Sam2Consus tool. Consensus sequences were then used as references used for variant analysis mapping using tanoti. Mapped files were used as input to diversi-tools and vnvs-tools. Data visualization was performed with an in-house *R* script using the tidyverse library and ggpubr.

Statistics

Statistical tests were performed using GraphPad Prism 9 with appropriate correction for multiple comparisons. *P<0.05, **P<0.01, ***P<0.001, ****P<0.0001 and no significance (n.s.) P>0.05.

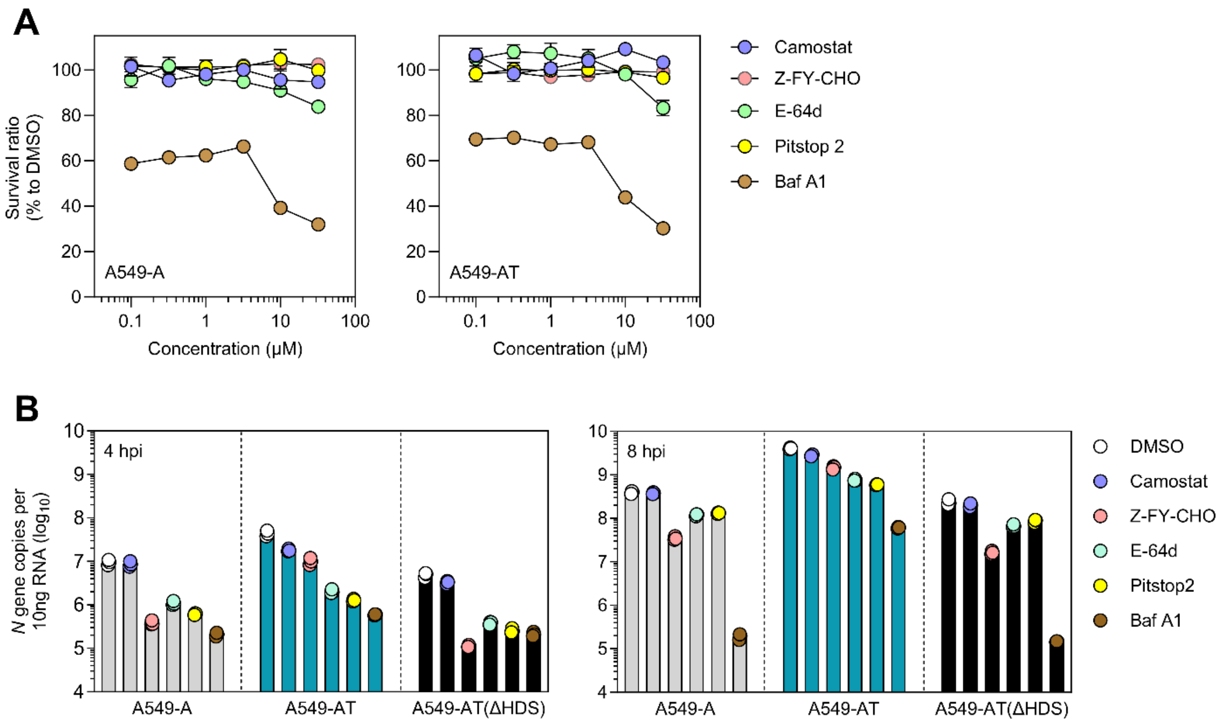

Fig. S1. Cell viability and entry inhibition upon drug treatment. (*A*) Cell viability of A549-A and A549-AT cells at 24 hours post treatment with Camostat mesylate (10 μM) (purple), Z-FY-CHO (10 μM) (red), E-64d (10 μM) (green), Pitstop2 (10 μM) (yellow) and Baf A1 (1 μM) (brown), normalized to vehicle control (DMSO, 100%). (*B*) Intracellular viral *N* gene copies per 10ng total RNA after entry inhibitor treatment in the indicated cell-lines at 4 hpi (left panel) and 8 hpi (right panel). Values represent mean±SEM of three biological replicates.

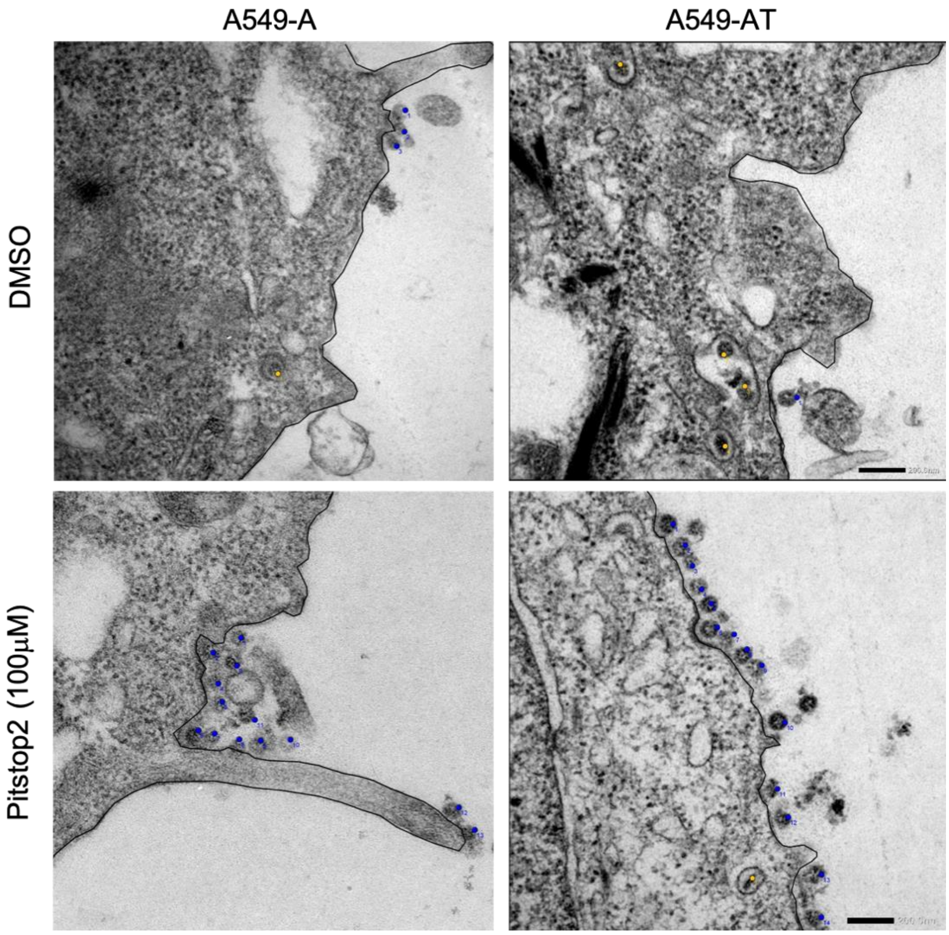

Fig. S2. Representative micrographs used for EM quantitation. EM images of A549-A and A549-AT cells after inoculation with B.1 virus (MOI 500) and treatment with either DMSO or CME inhibitor Pitstop2 (100μM) were acquired at 10 min after temperature switching to 37°C. Bar: 200nm.

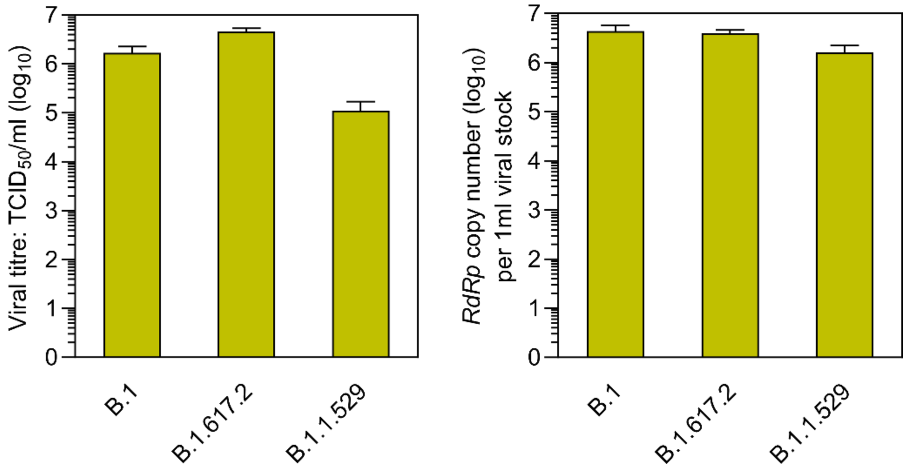

Fig. S3. Virus production from VeroE6 cells. Left panel: Infectious titres of B.1, B.1.617.2 and B.1.1.529 viruses produced in VeroE6 cells (MOI 0.01, 24 hpi). Values represent mean±SEM of two replicates. Right panel: *RdRp* copy numbers per mL determined for viral supernatants. Values present mean±SEM of three replicates.

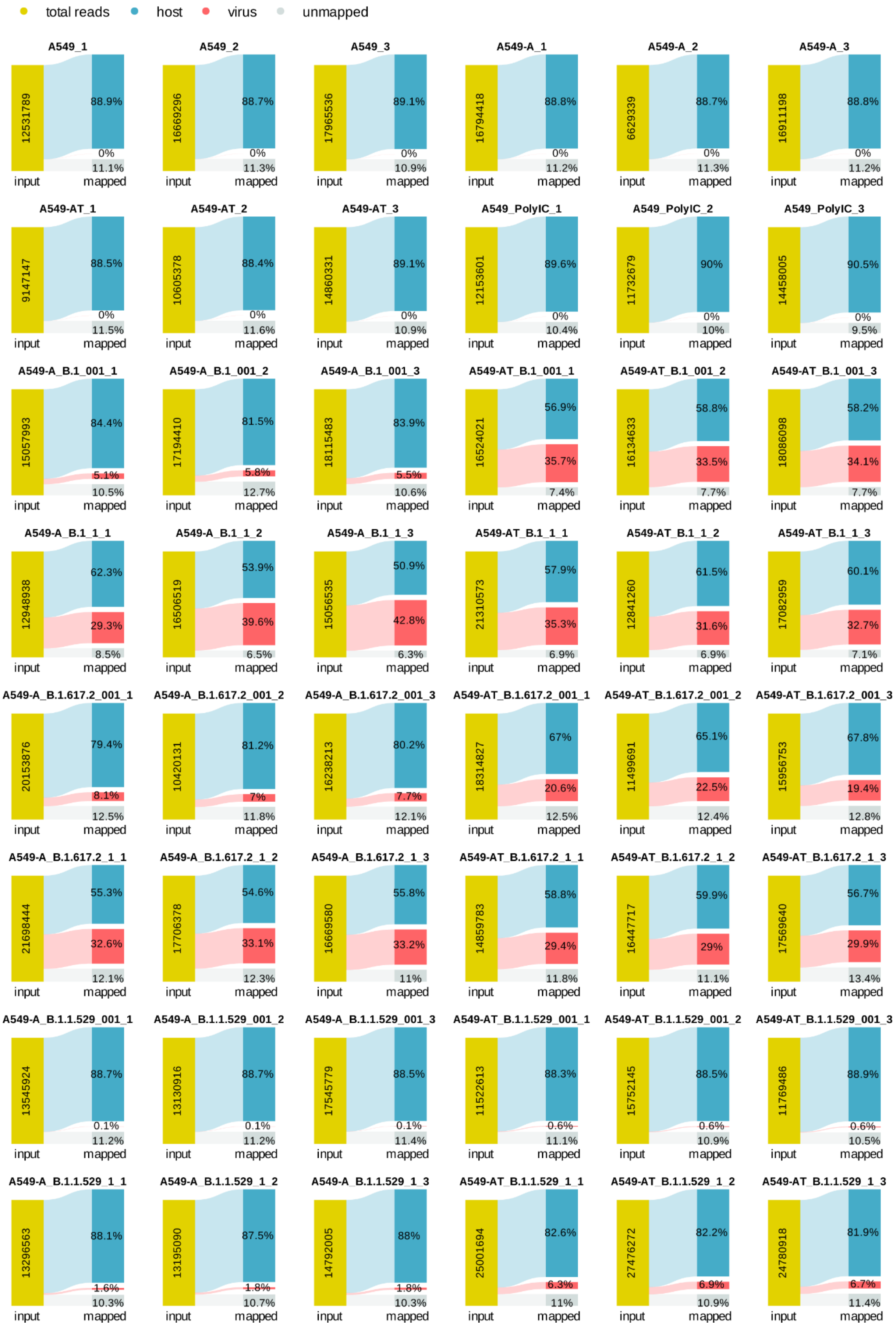

Fig. S4. Overview of RNA-seq data. Total input NGS reads for each sample were calculated (yellow), and further subdivided into host-mapped (blue), virus-mapped (red) and unmapped (grey). Percentage of total reads in each mapped category are associated with each plot.

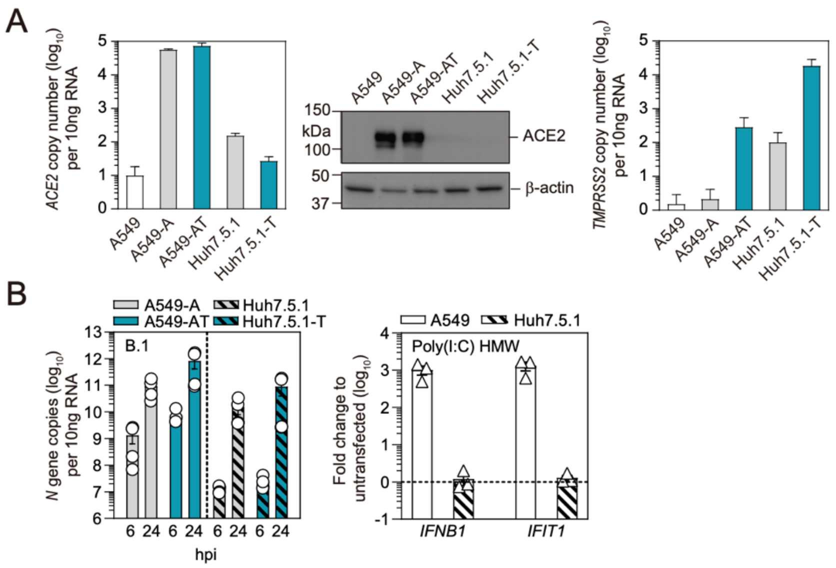

Fig. S5. ACE2 and TMPRSS2 expression, viral replication and interferon responses in A549 and Huh7.5.1 cells. (*A*) Left panel: absolute mRNA copy numbers for *ACE2* transcripts in 10 ng cellular RNA extracted from the indicated cell lines. Middle panel: ACE2 protein expression in the indicated five cell-lines. Right panel: absolute mRNA copy numbers for TMPRSS2 transcripts. (*B*) Left panel: *N* gene copies in the indicated cells infected with B.1 virus at 6 and 24 hpi. Right panel: *IFNB1* and *IFIT1* induction in A549 and Huh7.5.1 cells transfected with high-molecular weight Poly (I:C) for 24 h. Values represent mean±SEM of three technical replicates.

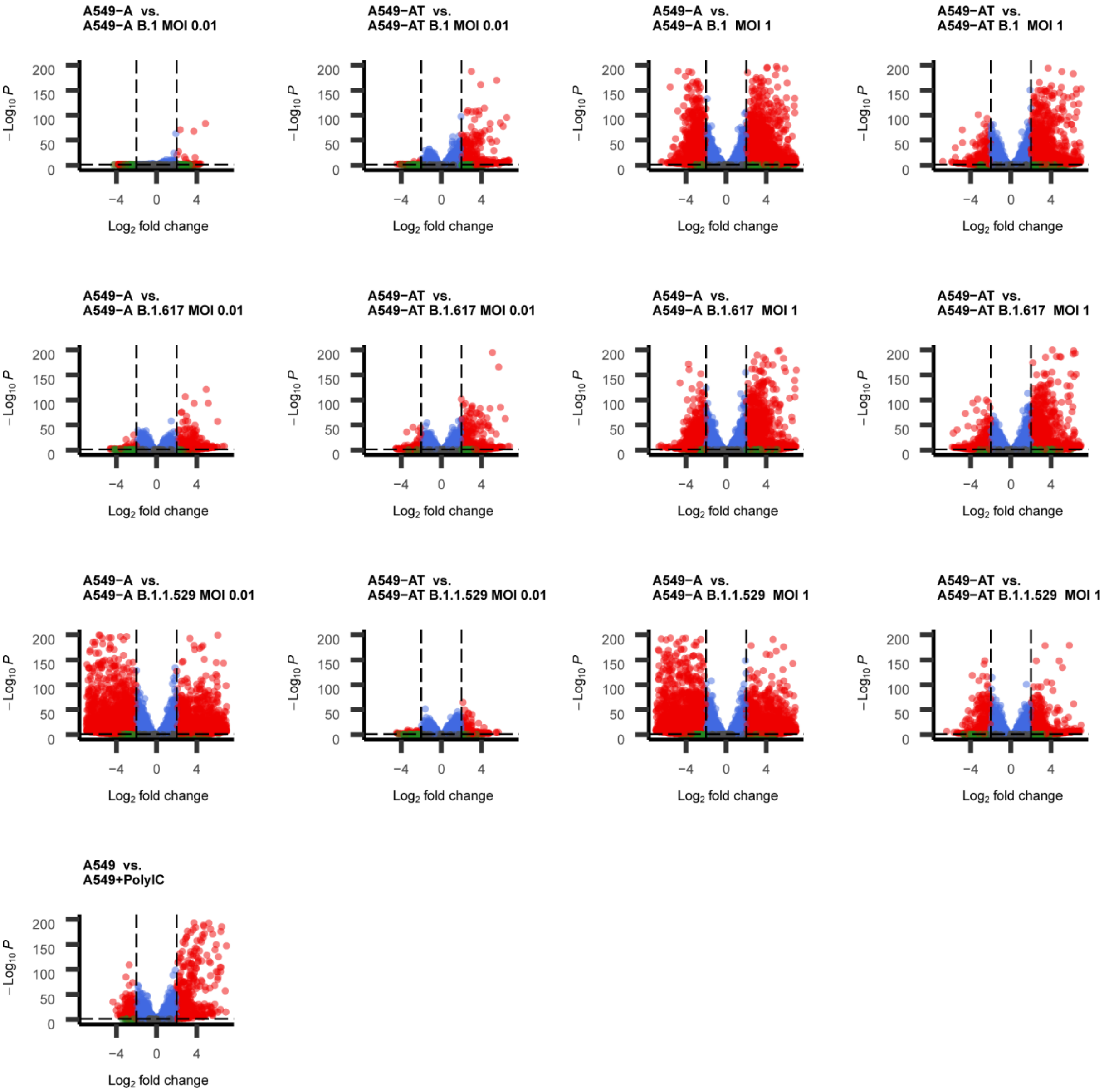

Fig. S6. Volcano plots visualize differentially expressed genes induced upon SARS-CoV-2 infection or Poly(I:C) treatment of engineered or parental A549 cells, respectively. Infection of the indicted cell-lines with viruses B.1 (top row), B.1.617.2 (second row) and B.1.1.529 (third row). Transfection of parental A549 cells with Poly(I:C) (bottom row). Left two columns: MOI 0.01; right two columns: MOI 1. Thresholds for FDR $\log_{10}P$-values (y-axes) and $\log_2$-fold change (x-axes) were 0.05 and 2, respectively. Genes exceeding thresholds are highlighted red

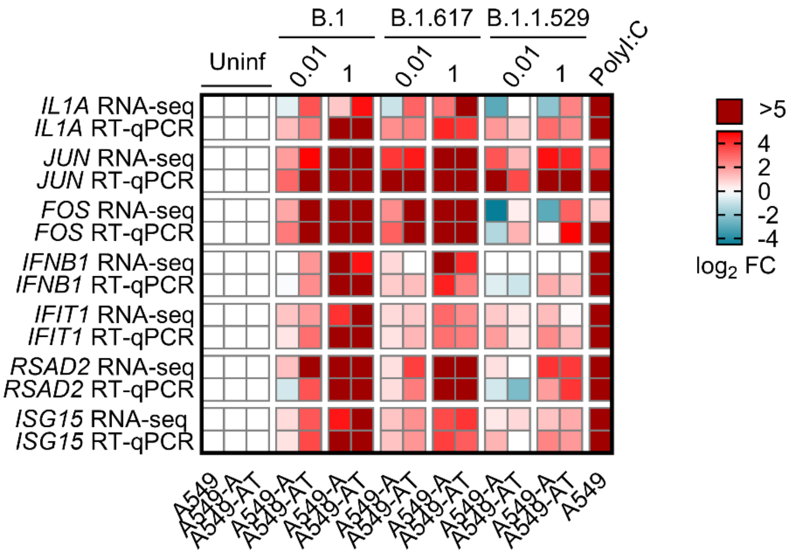

Fig. S7. Comparison of selected host gene induction by RT-qPCR and RNA-seq. Heat map visualizes fold change (log_2) in mRNA expression for seven selected host transcripts after SARS-CoV-2 infection of A549-A and A549-AT cells with the indicated virus, or transfection of A549 cells with Poly(I:C). For both RNA-seq and RT-qPCR, fold change is relative to expression levels in the respective uninfected control cell-line and represents mean values from N=3 biological replicates.

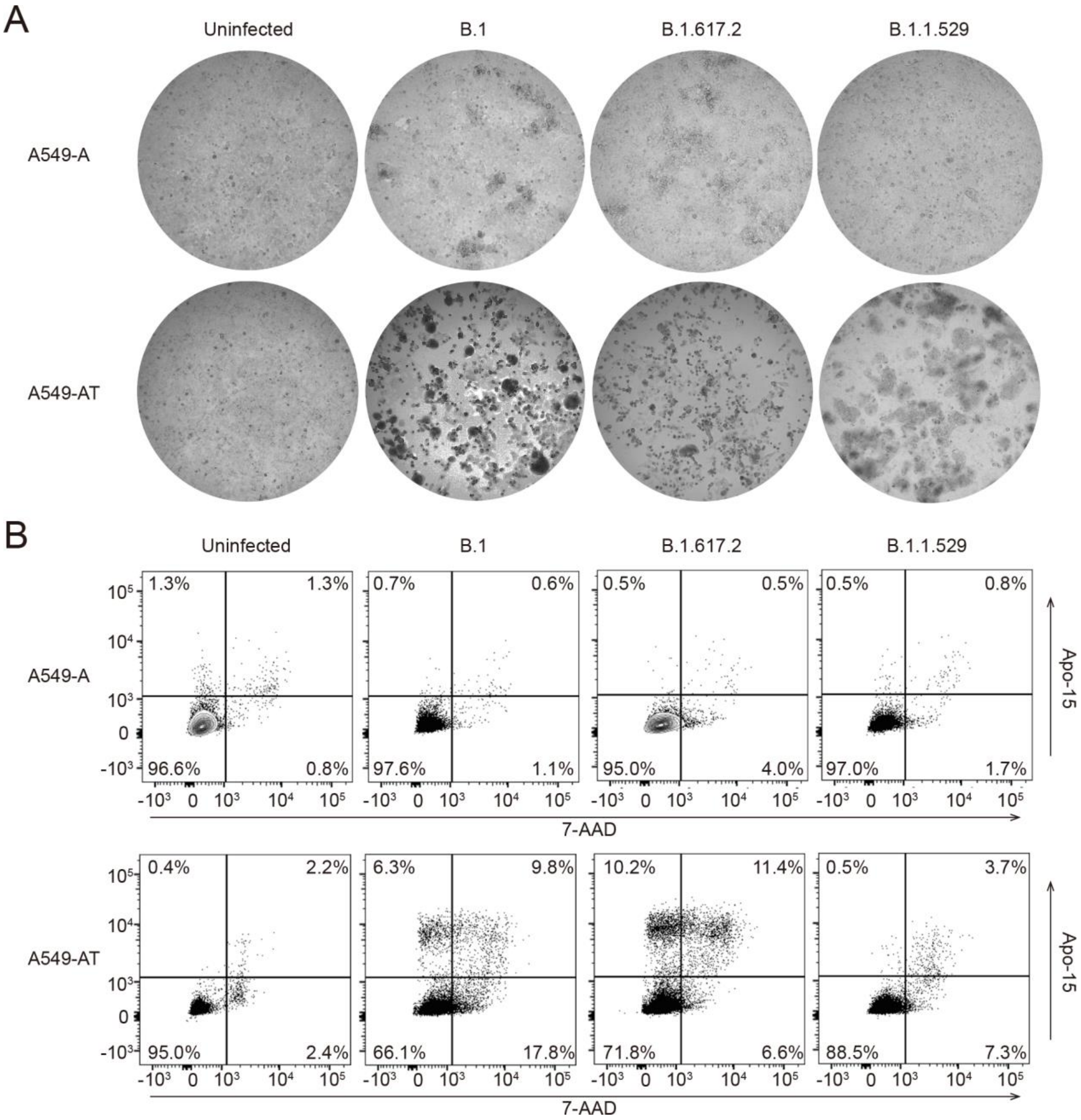

Fig. S8. Virus replication-induced cell-death. (*A*) A549-A and A549-AT cells were left uninfected or infected with B.1, B.1.617.2 and B.1.1.529 viruses at MOI of 0.01. At 72 hpi, phase contract images of cells were taken. (*B*) Apotracker green/7-AAD FACS plots. Single-cell suspensions of the above cells were prepared, fixed and subjected to an Apotracker/7-AAD co-staining, followed by flow cytometry. Images are representatives of three independent experiments.

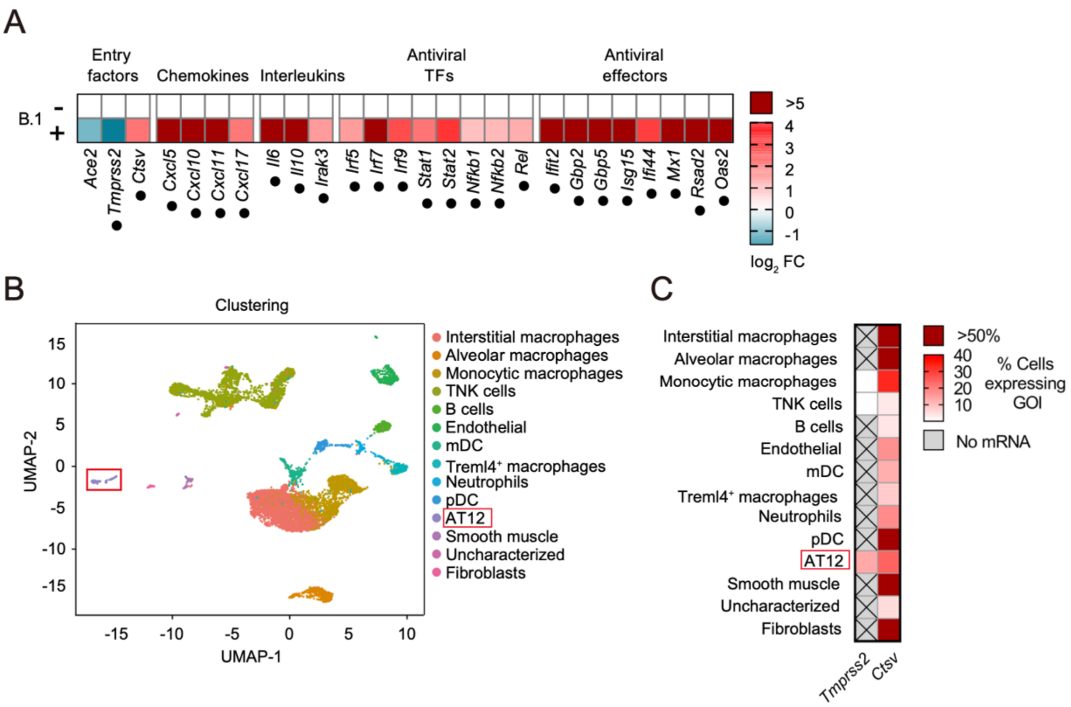

Fig. S9. *Tmprss2* transcripts are depleted upon SARS-CoV-2 infection and expressed in hamster pulmonary AT12 cells. (*A*) Heat map visualizes mean fold-change in lung resident mRNA expression (log_2) for selected transcripts after SARS-CoV-2 infection of hamsters (N=4), relative to expression levels in uninfected control hamsters (N=4). ● = FDR P<0.05. (*B*) UMAP plot reveals lung resident cell types in N=4 hamsters. AT12 cells are boxed in red. (*C*) scRNA-seq data showing *Tmprss2* and *Ctsv* cell type-specific expression in lung resident cells. AT12 cells are boxed. GOI: gene of interest.

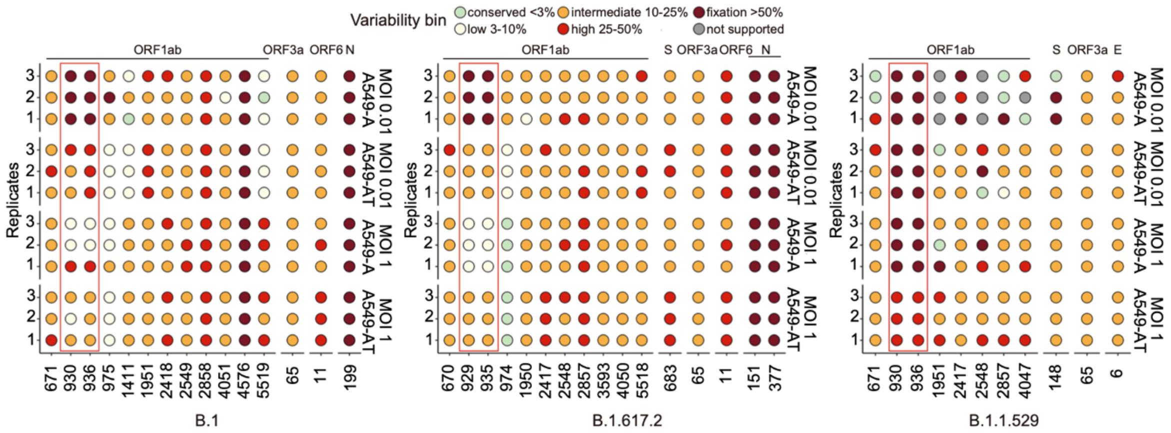

Fig. S10. Viral mutation frequencies above 3%. Dot plots highlight amino-acid variants >3% frequency in viral populations, relative to the reference strain sequences for B.1 (left), B.1.617.2 (middle) and B.1.1.529 (right). For each plot, biological replicates are displayed on the left y-axis and cell-line/MOI are presented on the right y-axis. The location of variable sites is displayed on the x-axis as amino acid numbering, with viral proteins in which they are located labeled above. For each position in the dot plot, the frequency of variability at that position is colored-coded relative to the key positioned above. Changes in the amino acid consensus (>50% frequency) are highlighted in dark red. Orf1a/1b residues 930/929 and 936/935 are highlighted by red boxes.

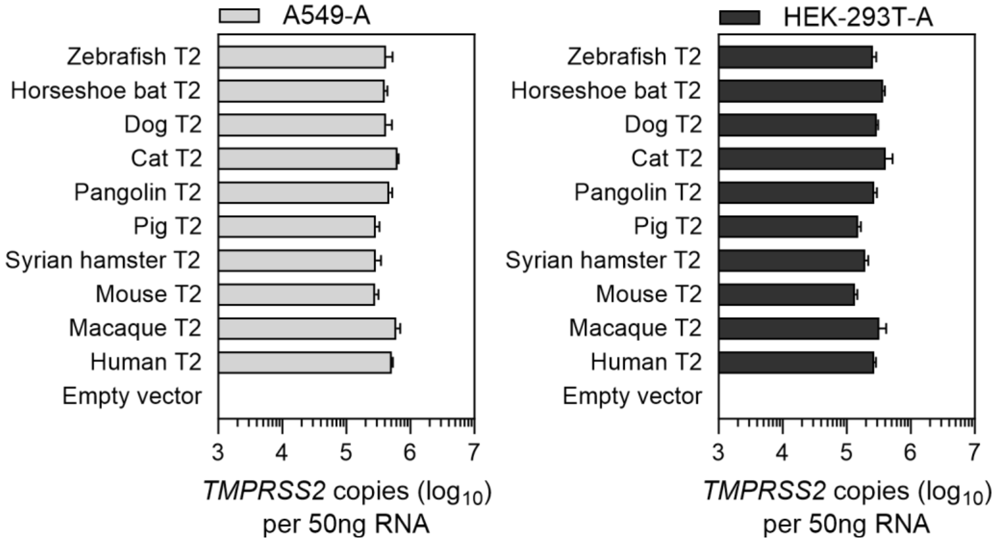

Fig S11. Quantification of *TMPRSS2* orthologue expression. Absolute mRNA copy numbers for *TMPRSS2* transcripts from the indicated species in 50 ng cellular RNA extracted from pseudotyped particle transduced A549-A (left) or HEK-293T-A cells (right).

SI References

1. Wilhelm A, *et al.* (2022) Limited neutralisation of the SARS-CoV-2 Omicron subvariants BA.1 and BA.2 by convalescent and vaccine serum and monoclonal antibodies. *EBioMedicine* 82:104158.
2. Schindelin J, *et al.* (2012) Fiji: an open-source platform for biological-image analysis. *Nat Methods* 9(7):676-682.
3. Blume C, *et al.* (2021) A novel ACE2 isoform is expressed in human respiratory epithelia and is upregulated in response to interferons and RNA respiratory virus infection. *Nat Genet* 53(2):205-214.
4. Miserey-Lenkei S, *et al.* (2021) A comprehensive library of fluorescent constructs of SARS-CoV-2 proteins and their initial characterisation in different cell types. *Biol Cell* 113(7):311-328.
5. Koutsoudakis G, *et al.* (2006) Characterization of the early steps of hepatitis C virus infection by using luciferase reporter viruses. *J Virol* 80(11):5308-5320.
6. Kumar S, Stecher G, & Tamura K (2016) MEGA7: Molecular Evolutionary Genetics Analysis Version 7.0 for Bigger Datasets. *Mol Biol Evol* 33(7):1870-1874.
7. Jumper J, *et al.* (2021) Highly accurate protein structure prediction with AlphaFold. *Nature* 596(7873):583-589.
8. Landau M, *et al.* (2005) ConSurf 2005: the projection of evolutionary conservation scores of residues on protein structures. *Nucleic Acids Res* 33(Web Server issue):W299-302.
9. Pettersen EF, *et al.* (2004) UCSF Chimera--a visualization system for exploratory research and analysis. *J Comput Chem* 25(13):1605-1612.
10. Kyte J & Doolittle RF (1982) A simple method for displaying the hydropathic character of a protein. *J Mol Biol* 157(1):105-132.
